# Biologically Inspired, Cell-Selective Release of Aptamer-Trapped Growth Factors by Traction Forces

**DOI:** 10.1002/adma.201806380

**Published:** 2019-01-07

**Authors:** Anna Stejskalová, Nuria Oliva, Frances J. England, Benjamin D. Almquist

**Affiliations:** 1 Department of Bioengineering, Imperial College London, London SW7 2AZ, UK; 2 Department of Bioengineering, Imperial College London, London SW7 2AZ, UK; Grup d’Enginyeria de Materials (GEMAT), Institut Químic de Sarri, Universitat Ramon Llull, Via Augusta 390, Barcelona 08017, Spain

**Keywords:** aptamers, biomaterials, biomimetics, controlled release, growth factor delivery, mechanobiology

## Abstract

Biomaterial scaffolds that are designed to incorporate dynamic, spatiotemporal information have the potential to interface with cells and tissues to direct behavior. Here, a bioinspired, programmable nanotechnology-based platform is described that harnesses cellular traction forces to activate growth factors, eliminating the need for exogenous triggers (e.g., light), spatially diffuse triggers (e.g., enzymes, pH changes), or passive activation (e.g., hydrolysis). Flexible aptamer technology is used to create modular, synthetic mimics of the Large Latent Complex that restrains transforming growth factor-*β*1 (TGF-*β*1). This flexible nanotechnology-based approach is shown here to work with both platelet-derived growth factor-BB (PDGF-BB) and vascular endothelial growth factor (VEGF-165), integrate with glass coverslips, polyacrylamide gels, and collagen scaffolds, enable activation by various cells (e.g., primary human dermal fibroblasts, HMEC-1 endothelial cells), and unlock fundamentally new capabilities such as selective activation of growth factors by differing cell types (e.g., activation by smooth muscle cells but not fibroblasts) within clinically relevant collagen sponges.

The applications for biomaterial scaffolds are numerous and range from stem cell biology[1] to disease modeling[2] to regenerative medicine.[3] There is a growing appreciation of the need to incorporate dynamic biological information into these scaffolds[4] in order to match the in vivo environment of the native tissue, where transient biological signaling is a fundamental aspect of tissue growth and repair: the establishment of both spatiotemporal biochemical and mechanical gradients guides embryonic morphogenesis[5] and coordinates the process of tissue repair.[6] This has spurred a push to design biomaterials that interface both in space and time with tissues.[7] Recent research has enhanced our ability to imbue scaffolds with temporal information that is transiently activated by stimuli such as light, enzymes, pH, complementary ligands, or an integrated combination of multiple stimuli.[2,3,8–15]

However, methods that provide powerful functionality in vitro, such as activation by light, have limited applicability in vivo due to significant scattering and absorption.[16] Enzymes such as matrix metalloproteinases (MMPs) can provide a level of autonomous temporal control deep in vivo, but there are limitations to the spatial selectivity due to the ability of MMPs to diffuse throughout the local extracellular matrix (ECM). Furthermore, there are significant challenges in decoupling the degradation of the scaffold due to MMPs from the release of growth factors to independently control changes to the scaffold’s mechanical properties and the rate of protein release. Therefore, an orthogonal method that utilizes direct, localized activation of bioactivity may provide a novel strategy for creating autonomous biomaterial scaffolds that have the potential to function as dynamic platforms both in model in vitro systems and within the body.

Looking to nature for inspiration, one can see that evolution has developed a unique strategy for enabling rapid activation of growth factors within tissues that is orthogonal to enzymatic cleavage and does not rely on cells that are resident in the area to produce them on-demand. During wound healing, the growth factor TGF-*β*1 is deposited in an inactive state throughout the ECM in a protein cage called the Large Latent Complex (LLC), which displays two copies of the integrin binding sequence RGD (Figure 1A).[17,18] As wounds heal, myofibroblasts exert mechanical forces to remodel and contract the ECM. Concurrently, these myofibroblasts attach to the LLC via the RGD sequences and use cellular traction forces to unfold it, thus releasing and activating TGF-*β*1 (Figure 1B). TGF-*β*1 stimulation then creates a feed-forward signaling loop that drives activation of additional contractile myofibroblasts to remodel and close the wound.[17] In doing so, the LLC cleverly transforms a mechanical stimulus into a diffusible biochemical signal. This method of using cellular traction forces to trigger the activity of growth factors is unique to TGF-*β*1 but is an evolutionarily conserved mechanism in species going back in the phylogenetic tree to deuterostomes (e.g., sponges, urchins),[18] suggesting a compelling benefit in the face of highly selective evolutionary pressure. Therefore, the LLC provides a unique approach for harnessing traction forces as a mechanical trigger that transiently activates dormant growth factor signaling, and has surprisingly been unexplored from an engineering perspective as a strategy for cells to autonomously activate bioactivity within biomaterial constructs.

Inspired by the LLC, we set out to develop a highly flexible, materials-based system that uses a cell’s ability to generate traction forces as a cell-based trigger to activate growth factors. The ideal system should be adaptable to any growth factor or cytokine of interest without the need for techniques such as protein engineering and should enable simple integration with any substrate or scaffold of interest. By providing a substrate-independent pendant nanostructure that is responsive to cellular traction forces, the system may act as a novel trigger to activate growth factors and be amenable to substrates and scaffolds relevant for both basic and translational in vitro and in vivo applications.

To facilitate the desired flexibility of the platform, we harnessed the unique properties provided by oligonucleotide aptamers. Aptamers are short, chemically synthesized singlestranded oligonucleotides that fold into three-dimensional structures and bind and inhibit proteins with affinities and specificities that rival antibodies.[19] They are discovered and optimized by an in vitro selection and evolution process called SELEX (Systematic Evolution of Ligands by Exponential Enrichment), making it theoretically possible to create aptamers that target virtually any protein of interest.[20,21] Importantly, the binding constant of the aptamer is dependent on its ability to fold into a structure that fits the relevant binding pocket on the targeted protein, and we hypothesized that direct application of cellular traction forces to the aptamer would trigger unfolding, eliminating the binding affinity and releasing and activating the bound protein (Figure 1C,D). Given that aptamers commonly have a *K*_D_ on the order of sub-nanomolar to sub-picomolar,[22] the potential to actively modulate the binding strength by ten to twelve orders of magnitude has the unique potential to significantly reduce nonspecific release while simultaneously facilitating the delivery of soluble, diffusible proteins.

In order to enable the use of traction forces to trigger the unfolding of the aptamers and simultaneous release of the bound proteins, we synthesized aptamers in which one end of the oligonucleotide is attached to a cell-adhesive peptide (e.g., the integrin binding peptide G**RGD**SPC), and the other end has a chemical group that facilitates facile conjugation to any substrate/scaffold of interest (e.g., thiol) (Figure 2A,B; Figures S1–S5, Supporting Information). We call these structures TrAPs: **Tr**action Force-**A**ctivated **P**ayloads. In agreement with prior research using DNA tension probes,[23,24] primary human dermal fibroblasts (HDFs) (Figure 1C,D) and HMEC-1 endothelial cells (Figures S6 and S7, Supporting Information) attach to surfaces coated with TrAPs, whereas TrAPs that contain R**D**G, the scrambled version of the integrin-binding peptide RGD that is not recognized by integrins, display no surface attachment (scr-TrAPs). To demonstrate the flexibility of the strategy, we synthesized TrAPs for both PDGF-BB[25] and VEGF-165[26] using previously reported aptamers with *K*_D_ values of 100 and 200 pM, respectively (Table S1, Supporting Information). Previous literature has demonstrated that an aptamer’s *K*_D_ correlates strongly with the level of nonspecific leakage.[27] In our hands, TrAPs display minimal nonspecific leakage following thorough washing, suggesting that the TrAP-specific modifications do not significantly affect the nonspecific release behavior (Figure 3A).

Having verified that cells recognize and adhere to unloaded TrAPs and that there is minimal nonspecific leakage of growth factors, we set out to validate the functional ability of the TrAP platform to impact cellular behavior. In the first instance, to prove the ability of cells to activate growth factors bound to TrAPs, we used a straightforward method to determine whether PDGF-BB TrAPs can increase the proliferation of HDFs, which we verified to have a dose-dependent response to PDGF-BB (Figure 3B).

We started by functionalizing 2D coverslips with RGD/RDG, PDGF-BB TrAPs and RGD/RDG, or PDGF-BB scr-TrAPs and RGD using a routine maleimide-thiol click reaction (Figure 3C; Figures S8 and S9, Tables S2 and S3, Supporting Information). Titration with RDG/RGD across samples was done to ensure that any differences in proliferation were due to differences in available growth factor concentrations and not due to variable RGD concentrations (Figure 2C,D). Serum-starved HDFs were seeded on substrates that were both unloaded and preloaded with PDGF-BB and compared to RGD coverslips with and without soluble PDGF-BB. Metabolic activity at 48 h after seeding was used as a measure of proliferation. PDGF-BB-loaded TrAP-modified surfaces demonstrated significantly higher proliferation compared to both PDGF-BB-loaded and unloaded scr-TrAPs, and unloaded PDGF-BB TrAPs (Figure 3D).

These proof-of-concept data provide critical evidence that the release and activation of PDGF-BB is due to RGD-mediated interactions. As expected, the soluble PDGF-BB samples demonstrated significantly higher proliferation than the PDGF-BB-loaded TrAPs, in agreement with previous studies using surface-immobilized growth factors.[28] Furthermore, research using molecular tension probes on 2D surfaces has demonstrated that the transduction of force occurs locally at the periphery of the cell,[24] with approximately 5% efficiency in activating traction probes at the edges of cells and close to 0% efficiency at the central part of the cell.[23] This data suggests that only a small fraction of the 1.5 ng of surface-bound PDGF-BB in TrAPs is activated at any given time, making the level of PDGF-BB stimulation significantly lower for TrAP-functionalized surfaces than for freely diffusible PDGF-BB. Similar increases in proliferation were seen with endothelial cells on 2D polyacrylamide gels functionalized with VEGF TrAPs via UV light (Figures S10–S12, Supporting Information), demonstrating the flexibility and adaptability of TrAP function to different cell types, growth factors, substrates, and conjugation strategies.

Having verified the functionality of TrAPs on planar culture surfaces, we next set out to evaluate the functionality of TrAPs within 3D collagen sponges. To create TrAP-functionalized collagen, we used a two-step functionalization approach that simultaneously cross-linked the collagen sponges via EDC/NHS while also attaching pendant maleimide groups (Figure S13, Supporting Information). Following cross-linking, we conjugated TrAPs with 5′ terminal thiols to the sponges. Successful incorporation of maleimides and TrAPs was validated using both FAM-labeled RGD and fluorescently tagged antisense DNA (Figures S13 and S14, Supporting Information).

Sponges functionalized with PDGF-BB TrAPs or scr-TrAPs were loaded with PDGF-BB and washed to remove excess growth factor. Following washing, serum-starved HDFs were seeded on the sponges, and proliferation measured 96 h later and compared to cross-linked collagen sponges with and without soluble PDGF-BB. In agreement with planar surfaces, collagen functionalized with TrAPs displayed significantly higher proliferation than collagen functionalized with scr-TrAPs and collagen with no growth factor (Figure 3E). However, unlike the 2D experiment, the proliferation in collagen sponges functionalized with TrAPs was statistically similar to the soluble PDGF-BB and TrAPs displayed the largest maximal increase in proliferation. This difference may be due to a more concentrated presentation of PDGF-BB around the cells in 3D compared to the freely diffusible growth factor. Alternatively, the 3D environment may lead to a higher level of TrAP activation around the HDFs. In either case, this proof-of-concept data suggests that TrAPs promote proliferation in 3D environments at least as well as soluble growth factors.

While many strategies have been developed to successfully imbue 3D scaffolds with growth factors, such as covalent cross-linking and heparin binding,[29] the use of traction forces as a trigger, along with the modular nature of the TrAPs, should provide unique abilities for controlling the activation and release of bound growth factors. Therefore, following the successful confirmation that traction forces can be used to activate growth factors across 2D and 3D environments, we next aimed to demonstrate that TrAPs enable fundamentally new capabilities when compared to all existing strategies for engineering triggered release. Specifically, we hypothesized that by careful selection of the cell-adhesive peptide, it should be possible to create TrAPs that are selectively activated by cell types that express the correct adhesion receptor for the chosen peptide. In doing so, TrAPs would enable the new ability to link the local activation of growth factors with the transient arrival of a defined type of cell within the scaffold. To evaluate this ability, we first screened two different adhesive peptides, REDV (binds *α*_4_*β*_1_ integrin) and VAPG (binds a 67 kDa nonintegrin adhesion receptor on smooth muscle cells),[30] for their ability to promote adherence of either fibroblasts or primary human smooth muscle cells (SMCs). Data from functionalized coverslips found that both cell types attach to RGD and REDV, whereas only SMCs attach to VAPG (Figure 4A, Figure S15, Supporting Information).

This adhesion data confirms that the VAPG peptide enables selective adhesion of SMCs, although it does not allow for evaluation of whether adhesion and force transmission through a nonintegrin receptor will occur in biologically relevant scaffolds that contain competitive integrin-binding peptides (e.g., the GFOGER peptide in collagen, which binds *α*_1_*β*_1_, *α*_2_*β*_1_, *α*_10_*β*_1_, and *α*_11_*β*_1_ integrins[31]). To test this, we functionalized collagen sponges with either RGD-TrAPs, VAPG-TrAPs, or scr-TrAPs for PDGF-BB (Figure S16, Tables S2–S4, Supporting Information), seeded them with either HDFs or SMCs, and allowed the cells to grow in culture for up to two weeks (Figure 4B–F). As can be predicted from the 2D adhesion data (Figure 4A), there are significantly more SMCs present in scaffolds functionalized with either RGD-TrAPs or VAPG-TrAPs compared to scr-TrAPs and scaffolds functionalized with only maleimides (Mal-Collagen) after one week, whereas scaffolds with RGD-TrAPs have more HDFs than VAPG-TrAPs (p = 0.051) (Figure 4C,E). Furthermore, SMCs on both scaffolds and HDFs on scaffolds with RGD-TrAPs display healthy elongated cell morphologies, in contrast with the HDFs on scaffolds with VAPG-TrAPs that are more rounded (Figure 4C). These trends are maintained after two weeks, with significantly more SMCs in scaffolds functionalized with either VAPG-TrAPs or RGD-TrAPs than scr-TrAPs and Mal-Collagen, whereas there are significantly more HDFs in scaffolds functionalized with RGD-TrAPs than those with either VAPG-TrAPs, scr-TrAPs, or Mal-Collagen (Figure 4D,F). Based on the microscopy images (Figure 4C,D), there does not appear to be significant degradation of the cross-linked collagen sponges over two weeks for all conditions, which is in agreement with findings from previous studies.[32,33]

In addition to demonstrating the unique ability to enable cell-selective activation of growth factors, these data also establish that: (i) TrAPs are stable and able to maintain increased cell numbers for at least two weeks in culture, including four growth factor-free media changes, and (ii) they do not exhibit the nonspecific leakage of growth factors at a level that results in a measurable increase in cell proliferation, which will occur when using aptamers or other affinity ligands with weaker binding affinities.[27,34] Both degradation of the TrAPs and nonspecific release will result in the activation of the bound growth factor; however, the demonstration that scr-TrAPs loaded with growth factors, which cannot be mechanically activated, result in virtually identical results to collagen without any TrAPs supports the ability of the TrAP platform to actively facilitate on-demand release of growth factors by cells.

In general, the fundamental nature of cells and tissues provides a significant challenge when designing materials that seamlessly bridge the biotic–abiotic interface; by continually integrating transient cues from their surroundings such as signaling proteins and matrix compliance, these biological systems make adaptive decisions that guide their behavior.[35] This has led to a push to develop biomaterial systems that incorporate both spatial and temporal information to transiently modulate cell–material interactions.[3,9,36] However, there are a lack of technologies that enable on-demand activation of bioactivity via direct interaction with cells via their innate interactions with materials, as opposed to exogenous intervention via strategies such as light. The data presented here form the basis of a new method for integrating latent growth factor signaling within a wide range of biomaterial-based systems that is activated by direct interaction with cells. By identifying cellular traction forces as an innate, but until now overlooked, stimulus for triggering growth factor activity, this research opens new possibilities in designing dynamic biomaterial systems.

In the past it has been suggested that for biomaterials of the future, “understanding the way in which complex dynamic behaviours are accomplished in nature may lead to the design of novel materials that mimic nature, not through presenting active motifs replicated exactly from biological molecules, but rather through reproducing the functional behaviour of these biological materials to obtain properties that are currently unavailable.”[37] TrAPs fully embrace this approach; mimicking the functional ability of the LLC via a flexible, fully synthetic system enables the use of cellular traction forces as a mechanical trigger, providing a fundamentally new approach to controlling the activation and delivery of growth factors within 3D environments,[29,36] along with unique benefits, such as cell-selective activation, that are not possible via any existing methods. Critically, the data presented here demonstrate the easy adaptability of TrAPs to multiple platforms (coverslips, polyacrylamide gels, collagen sponges), cell types (endothelial cells, fibroblasts, smooth muscle cells), immortalized cell lines and primary cells, and growth factors (PDGF-BB, VEGF). Additionally, due to the pendant, modular nature of the platform, we predict it will be straightforward to integrate TrAPs into a variety of established fabrication workflows, including both photopatterning and 3D printing, enabling synergistic integration with the newest advances for synthesizing three-dimensional biomaterials.

Importantly, by designing TrAPs to harness the bioinspired approach of using mechanical activation provides an orthogonal method to other endogenous triggers such as enzymatic cleavage, and can function in places where exogenous activation via light is not possible. Furthermore, TrAPs require the transmission of force through the aptamer, which decouples the mechanical activation of TrAPs from the mechanical force transmitted through the underlying scaffold, positioning the platform as a compelling technology for applications involving sites of significant mechanical deformation (e.g., heart). The use of aptamers also provides a high level of growth factor selectivity via an affinity-based approach,[38] which is not possible through the use of more promiscuous strategies where the binding domains are derived from various components of the ECM.[39,40] Additionally, the ability to abrogate binding affinity via force enables the use of high-affinity aptamers (sub-nanomolar affinities) that prevent significant nonspecific release. These features create intriguing possibilities for multiplexing the on-demand delivery of multiple, well-defined growth factors in order to facilitate synergistic signaling.

Building on the capability to enable cell-selective activation may provide a pathway for creating transiently activated autocrine and paracrine growth factor signaling based on the presence or absence of the targeted cell types. If designed to target the temporally coordinated arrival of different cells during tissue repair,[41] this may enable the creation of temporally coordinated signaling via bidirectional cell–material interactions. Given that 99% of prior research utilizes only three cell adhesive peptides (RGD: 89%, IKVAV: 6%, YIGSR: 4%),[30] there remains significant untapped potential to push the use of novel adhesive peptides to advance this cell-selective capability. Finally, the ability to maintain increased cell numbers for at least two weeks in culture (Figure 4B,D,F) suggests that the aptamers may be stabilizing the growth factors against inactivation. This hypothesis is supported by prior research demonstrating that aptamers possess the ability to stabilize proteins against environmental stresses,[42] along with research showing that growth factors are stabilized and protected when bound to the ECM.[43]

Going forward, there is significant potential to build on these results for use during in vivo applications. Notably, the TrAPs used in this study do not display signs of significant degradation within two weeks when coupled to a matrix (Figure 4, Figure S17, Supporting Information), in agreement with previous studies on aptamer-functionalized hydrogels,[44] along with no observable degradation of unconjugated, soluble TrAPs over one week in primary fibroblast-conditioned media at 37 °C (Figure S18, Supporting Information). The DNA aptamers used here do not contain any modifications to increase nuclease resistance, other than the inherent changes to the 3′ and 5′ ends; in the past, these modifications have been shown to reduce exonuclease activity.[45] Additional modifications, such as the use of phosphorothioate bases, flipped bases, locked nucleic acids (LNAs), Spiegelmers (mirror-image L-oligonucleotide aptamers), and g-quadruplex aptamers can additionally improve the resistance to both exo- and endonuclease degradation.[45–47] Coupled with the prediction that there is likely steric protection of the oligonucleotide from enzymatic attack due to the presence of a bound protein and close association with the matrix, the TrAP platform provides significant potential to allow persistence in tissue microenvironments actively undergoing repair.

This ability to tune the stability of aptamers opens exciting possibilities for translational applications. Currently, there are a handful of aptamers going through clinical trials for inhibiting growth factor activity in pathological conditions.[19] These aptamers have already been optimized for stability in vivo and bind growth factors that are relevant for applications in tissue repair including VEGF (Bausch+Lomb), PDGF-BB (Ophthotech Corp.), CXCL12 (NOXXON Pharma), NGF (RIBOMIC Inc.), and FGF-2 (RIBOMIC Inc.) with high affinity. Through straightforward chemical modification of these aptamers to create TrAPs, they have significant potential to facilitate the controlled release of their targeted proteins for applications in therapeutic angiogenesis, nerve repair, bone repair, wound healing, and more. With numerous reports demonstrating the impact of controlled release of low dose growth factors, along with the synergistic benefits of combinatorial growth factor therapies,[29,48] TrAPs provide an enticing, fully synthetic method for integrating the controlled release of growth factors into a wide range of existing clinical biomaterials ranging from macroporous sponges to minimally invasive injectable materials.

In summary, the TrAP platform establishes a new strategy for controlling the activation and release of growth factors by relying on the direct application of cellular traction forces. The ability to target a vast array of proteins via aptamers; the ability to use native, unmodified proteins with full post-translational modifications (e.g., glycosylation); an accessible path to multiplexing with different TrAPs; the potential to facilitate synergistic integrin–growth factor crosstalk[49]; the ability to enable selective activation of growth factors by different cell types; and straightforward integration with virtually any surface or scaffold—all via a direct, fully synthetic materials-based approach—create a compelling list of features. As such, these data launch a fundamentally new method for harnessing traction forces as a biophysical trigger to activate localized bioactivity, creating new opportunities to create dynamic biomaterials for studies exploring fundamental biological phenomena as well as translational applications in regenerative medicine.[1–4,29]

## Experimental Section

Detailed experimental methods can be found in the Supporting Information.

## Supplementary Material

Supporting Information is available from the Wiley Online Library or from the author.

Supporting Information

## Figures and Tables

**Figure 1:**
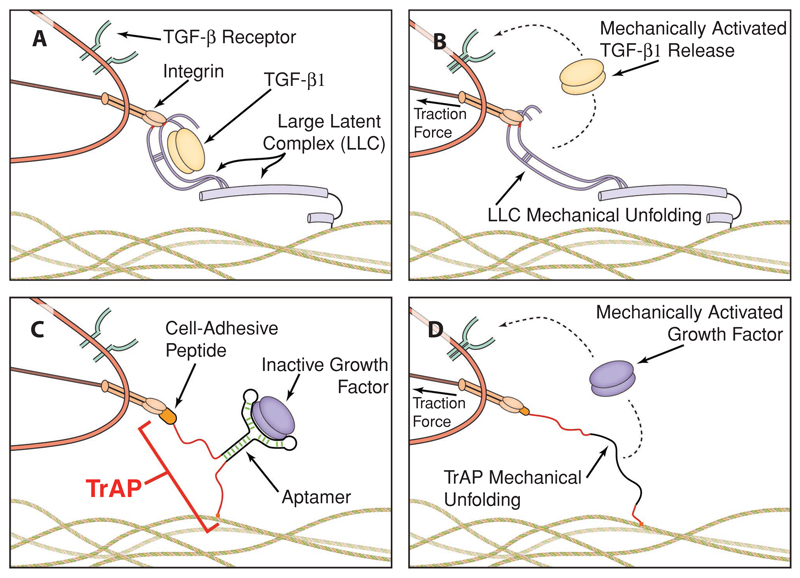
Bioinspired aptamers enable the creation of synthetic mimics of the natural TGF-*β*1 large latent complex (LLC). A,B) TGF-*β*1 LLC is deposited throughout the extracellular matrix. Upon application of cellular traction forces, the LLC unfolds, releasing and activating TGF-*β*1. C,D) By attaching a cell-adhesive peptide to one end of a nucleic acid aptamer and a cross-linking site to the opposite end for matrix conjugation, a flexible strategy for fully synthetic mimics of the LLC that are amenable to virtually any protein is possible. These bioinspired nanostructures are called TrAPs: **Tr**action Force **A**ctivated **P**ayloads.

**Figure 2:**
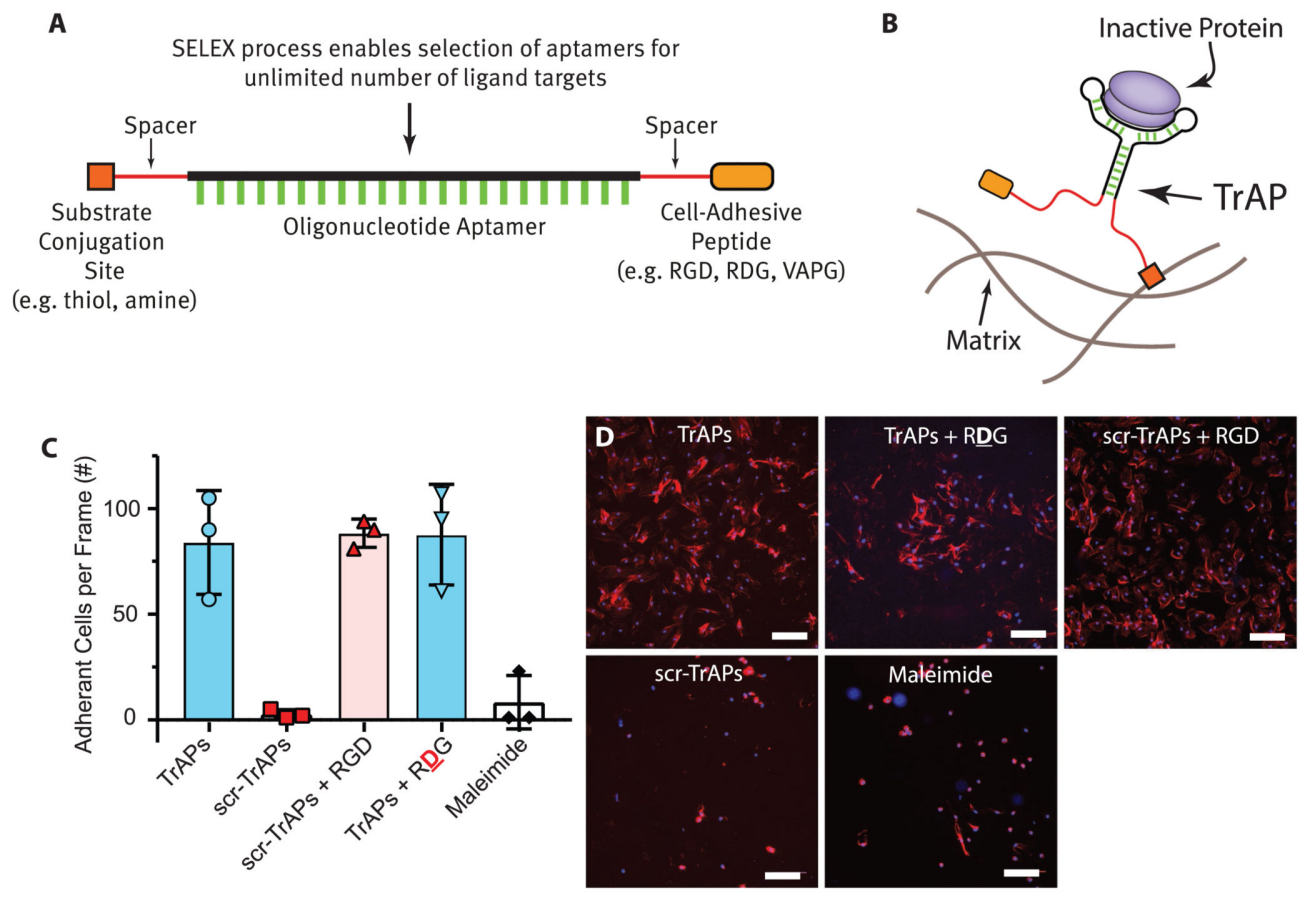
TrAPs are modular nanostructures that enable a high degree of design flexibility. A) TrAPs are aptamers with a cell adhesive peptide attached to one end and a chemical group for bioconjugation attached to the opposite end. Both end modifications can be easily changed depending on the specific application requirements. B) TrAPs adopt a folded structure when binding and inhibiting ligands, making them susceptible to unfolding due to mechanical forces. C) HDFs adhere to TrAP functionalized coverslips but not to scr-TrAP or maleimide-functionalized coverslips. Titrating scr-TrAPs with GRGDSPC peptides restores HDF ability to bind to surfaces containing scr-TrAPs (*n* = 3, One-Way ANOVA, Tukey post-hoc). ** *p* ≤ 0.005. D) Representative images of data quantified in (C). Scale bar = 200 μm.

**Figure 3:**
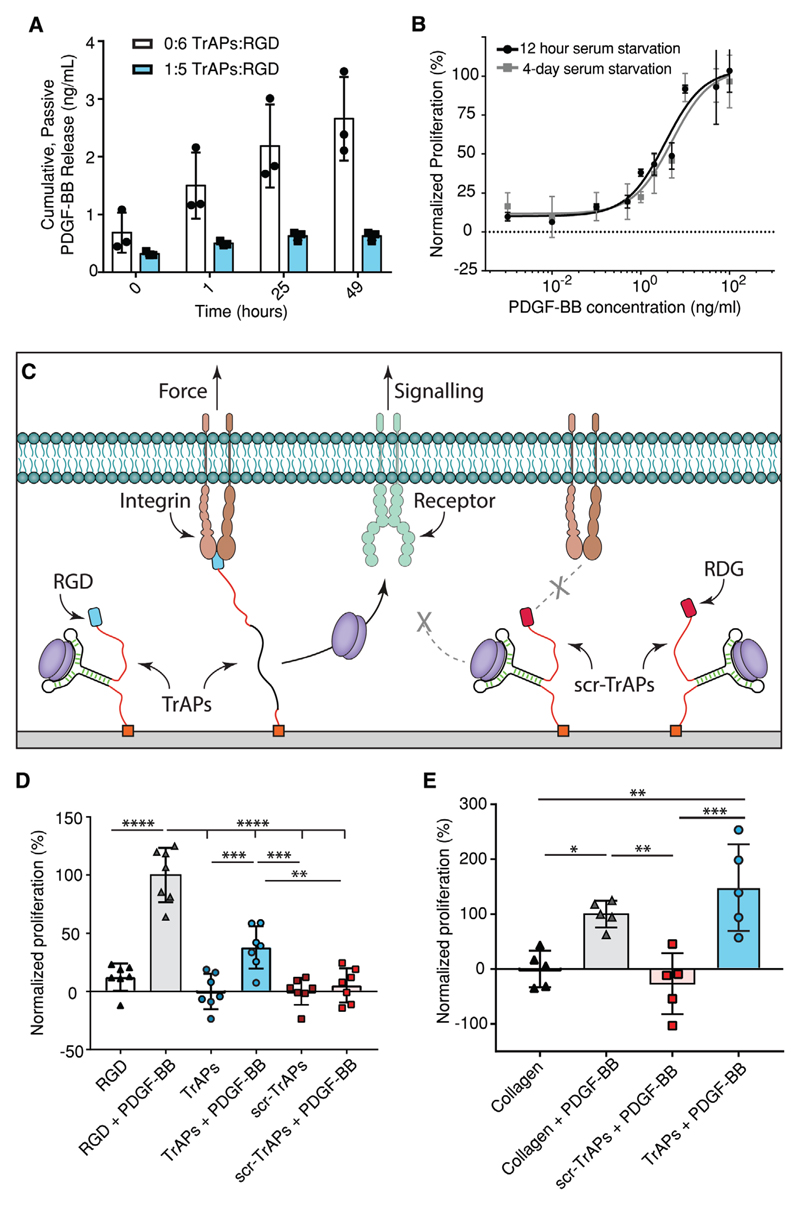
TrAPs enable the use of cellular traction forces as a trigger for activating PDGF-BB across 2D substrates and 3D scaffolds. A) TrAP-functionalized coverslips exhibit minimal growth factor release compared with RGD-functionalized coverslips, indicating that TrAPs are able to retain the ability to bind PDGF-BB (*n* = 3). B) Proliferation of HDFs increases in a dose-dependent manner (*n* = 4). C) The RGD peptide on TrAPs allows for traction force-mediated unfolding of the aptamer and subsequent release of the bound protein. Scr-TrAPs contain the non-integrin-binding peptide RDG and are unable to be recognized by integrins. D) HDFs on coverslips functionalized with PDGF-BB-loaded TrAPs proliferate more than on coverslips with unloaded TrAPs, unloaded scr-TrAPs, and scr-TrAPs loaded with PDGF-BB (*n* = 7). E) HDFs in collagen sponges functionalized with PDGF-BB-loaded TrAPs proliferate significantly more than HDFs in collagen sponges functionalized with scr-TrAPs loaded with PDGF-BB and RGD-modified collagen sponges without PDGF-BB (*n* = 5, One-Way ANOVA, Tukey post-hoc). *p ≤ 0.05, **p ≤ 0.01, ***p≤ 0.001, ****p ≤ 0.0001.

**Figure 4:**
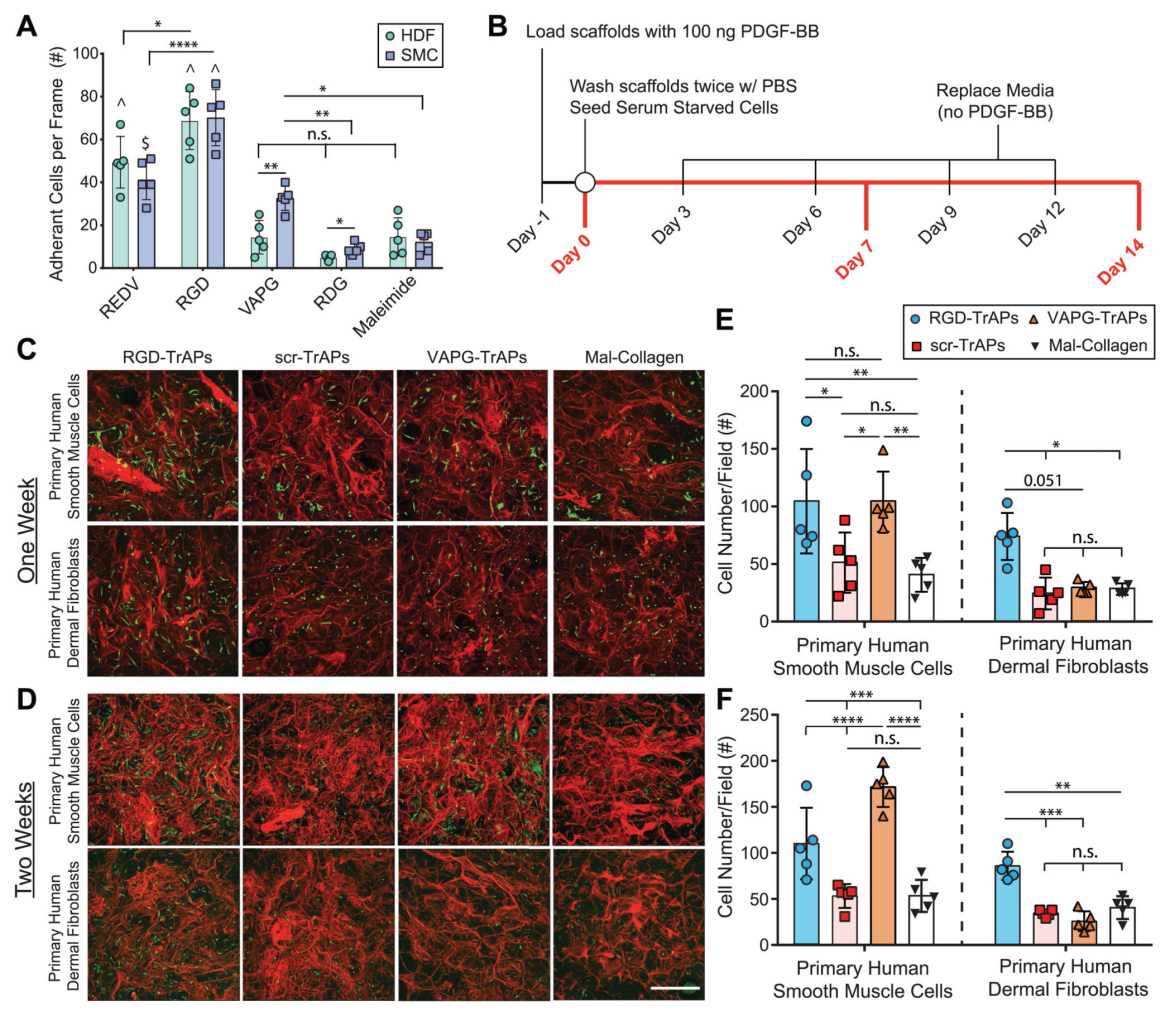
TrAPs enable selective activation of growth factors in 3D collagen scaffolds by different cell types based on the expression of adhesion receptors. A) SMCs, but not HDFs, can adhere to 2D glass coverslips functionalized with the peptide VAPG. B) Experimental protocol for one and two week studies of TrAP-functionalized collagen scaffolds. C–F) SMCs can activate PDGF-BB bound by either RGD-TrAPs or VAPG-TrAPs, whereas HDFs can only activate PDGF-BB bounded by RGD-TrAPs. Fluorescence images of collagen scaffolds after one (C) and two (D) weeks in minimal media (red: collagen; green: HDFs or SMCs). Quantification of cell numbers in scaffolds after one (E) and two (F) weeks. Scale bar = 500 μm (*n* = 5, One-Way ANOVA, Tukey post-hoc). *p ≤ 0.05; **p ≤ 0.005; ***p ≤ 0.0005; ****p ≤ 0.0001; ^*p* ≤ 0.0001 compared to VAPG, RDG, and maleimide. $*p* ≤ 0.0001 compared to RDG and maleimide.
