# Supplementary material for: Biologically Inspired, Cell-Selective Release of Aptamer-Trapped Growth Factors by Traction Forces
Source: Adv Mater. Author manuscript; Available in PMC 2019 Feb 14. (PMC6375388; doi:10.1002/adma.201806380)
Supplement: Supporting Information [file NIHMS81119-supplement-Supporting_Information.pdf]

Copyright WILEY-VCH Verlag GmbH & Co. KGaA, 69469 Weinheim, Germany, 2018.

ADVANCED MATERIALS

Supporting Information

for *Adv. Mater.*, DOI: 10.1002/adma.201806380

Biologically Inspired, Cell-Selective Release of Aptamer-Trapped Growth Factors by Traction Forces

*Anna Stejskalová, Nuria Oliva, Frances J. England, and Benjamin D. Almquist**

Copyright WILEY-VCH Verlag GmbH & Co. KGaA, 69469 Weinheim, Germany, 2019.

Supporting Information

Biologically Inspired, Cell-Selective Release of Aptamer-Trapped Growth Factors by Traction Forces

A. Stejskalová, N. Oliva, F. J. England, B. D. Almquist*

MATERIALS AND EXPERIMENTAL METHODS

Reagents and chemicals

Phosphoramidites compatible with UltraFAST deprotection (Bz-dA-CE, Ac-dC-CE, dmf-dG-CE, and dT-CE, LINK), 3'-Thiol-Modifier C3 S-S CPG (LINK, 2361), 5'-Bromohexyl Phosphoramidite (Glen Research, 10-1946-xx), spacer-CE Phosphoramidite 18 (HEG, LINK, 2129-F100X1), sodium azide (Sigma, S2002), N,N-Dimethylformamide (DMF, Sigma, D4551), ammonium hydroxide (Sigma, 16748-250ML-F), methylamine (Sigma, 426466), Illustra NAP-25 columns (GE-Healthcare, 17-0852-01), Novex™ TBE-Urea denaturing Gels (15%, 10 wells, EC6885BOX, Thermo Fisher), 10/60 ssDNA ladder (IDT, 51-05-15-01), 1X SYBR® Gold Nucleic Acid Gel Stain (S11494, ThermoFisher), Dibenzocyclooctyne-maleimide (Sigma, 760668, MW= 427.45), Dimethyl sulfoxide (DMSO, Sigma, D8418), Novex® TBE Running Buffer (Thermo Fisher, LC6675), Novex™ TBE-Urea Sample Buffer (Thermo Fisher, LC6876), DBCO-amine linker (761540, Sigma), sulfuric acid (Fisher Scientific, S/9240/PB17), hydrogen peroxide (Fisher Scientific, H/1750/17), 3-Aminopropyl(diethoxy)methylsilane (Sigma, 371890), borate buffer (Thermo Fisher, 28384, BupH™ Borate Buffer Packs, pH 8.5), maleimide-PEG2-succinimidyl ester (Sigma, 746223), 3-(Trimethoxysilyl)propyl methacrylate (Sigma-Aldrich, 440159-100ML, 98%), Acrylamide solution (40% w/v, A4058-100mL, Sigma Aldrich), N,N'-Methylenbisacrylamide solution (2% in H_20, M1533-25ML, Sigma Aldrich), N,N,N',N'-Tetramethylethylenediamine (TEMED, 99%, T981-100ML, Sigma-Aldrich), ammonium persulfate (APS, >98%, A3678-25G, Sigma-Aldrich), HEPES (Sigma), TFA (302031, Sigma) ,Acrylate-PEG-Maleimide (ACRL-PEG-MAL-3400-500mg, MW=3400, Creative PEG Works), QuantiFluor® ssDNA system (Promega, E3190), BSA (OmniPur® BSA, Fraction V, ColdMerck, 2905), PDGF-BB DuoSet ELISA (DY220) and DuoSet ELISA Ancillary Reagent Kit 2 (DY008) were purchased from R&D Systems, Collagen sponges (Avitene Ultrafoam Collagen, 1050050, 8.5cm x 12.5 cm x 3mm), 1-(2-Aminoethyl)maleimide hydrochloride (Sigma, 56951, MW 254.16), N-Ethyl-N′-(3-dimethylaminopropyl)carbodiimide hydrochloride (EDC, Sigma, 03449), N-Hydroxysulfosuccinimide sodium salt (NHS, Sigma, 56485), PrestoBlue™ Cell Viability Reagent (Thermo Fisher, A13261), MCDB 131 medium (10372-019, Sigma, no glutamine), foetal bovine serum (FBS, Life Technologies, 10270-106), L-Glutamine (Sigma, G8540), Epidermal Growth Factor, hydrocortisone (Sigma, H0888) , Penicillin-Streptomycin (Pen/Strep, Sigma, P4333), Human VEGF-165 (Biolegend, 583706, carrier-free), Dulbecco's Modified Eagle Medium (DMEM, low glucose, Sigma, D5546), Smooth Muscle Cell Basal Medium 2 (Promocell, C22262), Human Aortic Smooth Muscle cells (Promocell, C12533), PDGF-BB (Biolegend, 577304, carrier-free), formaldehyde (Thermo Fisher, 28908), Triton X-100, Alexa Fluor™ 633 Phalloidin (ThermoFisher, A22284), purified

mouse anti-paxillin (BD Biosciences, 610051), Alexa Fluor® 488 goat anti-mouse IgG cross-adsorbed secondary antibody (ThermoFisher, A11001), Hoechst 33342 (ThermoFisher, H3570), VECTASHIELD antifade mounting medium (VECTOR laboratories, H-1000), CellTracker^{TM} Green CMFDA (5-Chloromethylfluorescein diacetate) Dye (Thermo Fisher, C2925), Texas Red-X, succinimidyl ester, mixed isomers (Thermo Fisher, T6134), μCLEAR® bottom were used in all experiments (Greiner, 655986), O.C.T. compound, mounting media for cryotomy (VWR, 361603E).

Modification of Peptides

GRGDSPC, GRDGSPC, CREDVW and CVAPG peptide sequences containing cysteine (98% purity, guaranteed TFA removal) were purchased from GenScript. The as-received peptides were stored in their freeze-dried form at -80°C until needed, when they were resuspended in either deionised water or coupling buffer at 20mM. Before conjugation, peptides were reduced using a Pierce^{TM} Immobilized tris(2-chloroethyl) phosphate (TCEP) disulfide reducing gel. As the reducing gel is acidic, the pH was increased by incubating the gel with coupling buffer (50 mM sodium phosphate pH 7.2 at 4 °C, 50 mM NaCl, 10 mM EDTA) for 10 minutes. The gel with buffer was centrifuged, the buffer carefully removed and peptides were added to the TCEP-gel for 30 minutes, upon which the mixture was centrifuged and the peptides recovered. Dibenzocyclooctyne (DBCO) was introduced onto the cysteine-containing peptides using a dibenzocyclooctyne-maleimide heterobifunctional linker (Fig. S1a). The hydrophobic DBCO-maleimide linker was resuspended in dimethyl sulfoxide at 20 mM. Once the peptides had been reduced, the coupling reaction (Fig S1a) was left to proceed at 1:1.5 molar ratio of the 20mM maleimide-DBCO to peptide-thiol, with the peptide in excess in the coupling buffer at room temperature for at least 2.5 hours. The products (**1a, 1b**) were subsequently analysed using an analytical LC-MS (Fig. S3) and purified by preparative reverse phase high-performance liquid chromatography (HPLC, Shimadzu Phenomenex) on a C18 Gemini NX column (150 mm x 21.2 mm, 5 μM particle size, 110 Å pore size) (Fig. S4a). A water-acetonitrile gradient was used (95% to 5% H_2O) with 0.1% trifluoroacetic acid (TFA) added to the mobile phase. The TFA was removed using a rotary evaporator. The product was subsequently freeze-dried and stored at -80°C until needed. The identity of the products was confirmed using matrix-assisted laser desorption/ionisation in a positive mode (micromass MALDI-TOF). 1 μL of sample dissolved in DI water was deposited onto 1 μL of α-cyano-4-hydroxycinnamic acid (CHCA) matrix (Fig. S4b).

DNA Aptamer Synthesis

Aptamer synthesis was either done in-house using a MerMade 6 Oligonucleotide synthesiser (Fig. S2a) or purchased from commercial DNA synthesis providers (Integrated DNA Technologies, ATDBio) (Table S2). Synthesising and purchasing aptamers was done to ensure the feasibility of commercially purchasing all key components of the TrAP platform, in turn confirming this method is transferable to labs lacking DNA synthesis capabilities.

For aptamers synthesised in-house, azide and thiol terminated oligos with PEG or polyT spacers were synthesised using solid-phase phosphoramidite chemistry and on-column chemical conversions. The synthesis scale was 1μmol, and all unmodified DNA phosphoramidites compatible with UltraFAST deprotection (Bz-dA-CE, Ac-dC-CE, dmf-dG-CE, and dT-CE) were purchased from LINK technologies. The 3' end was modified with thiol using the 3'-Thiol-Modifier C3 S-S CPG solid support, and the 5' end was

functionalized with bromine using a 5'-Bromohexyl phosphoramidite. The spacer-CE Phosphoramidite 18 or polyT tails (5 thymines per tail) were introduced as a penultimate modification on both ends. During the synthesis, standard manufacturer-recommended coupling times and dilutions were employed. The DMT-On setting was used to prevent the cleavage of bromine.

Following synthesis, the columns were rinsed with acetonitrile and the 5'-bromohexyl converted to 5'-azidohexyl using a mixture of 13mg of sodium azide and 30mg of sodium iodide in 2 mL of N,N-Dimethylformamide. The mixture was left to react for 1 hour at 65°C. Once cooled, the oligos were rinsed twice with DMF and acetonitrile. Subsequently, oligos were cleaved from the solid support and the protecting groups removed using aqueous ammonium hydroxide and methylamine (1:1) at RT for 2 hours. Finally, the deprotected oligos were purified using size-exclusion chromatography Illustra NAP-25 columns equilibrated with DI water.

The concentration of oligos was measured using a NanoDrop. The quality of synthesis was analysed using denaturing gel electrophoresis on Novex™ TBE-Urea denaturing gels and judged against a 10/60 ssDNA ladder (Fig. S5b). 1X SYBR® Gold Nucleic Acid Gel Stain in TBE buffer was used to stain nucleic acids based on manufacturer's instructions (20-minute incubation under agitation, protected from light) and the bands were visualised using a BioSpectrum® Imaging System (UVP). Images were captured at several exposure times. Analysis of the bands was performed using FIJI.

Fabrication of TrAPs for 2D and 3D experiments

100µM azide-terminated oligonucleotides **(2 and 4)** (Apt_PDGFBB_1 or Apt_PDGFBB_2, Table S2) were reacted with DBCO-functionalized peptides **(1a-b)** at 1:2 molar ratio for 24 hours at RT in DI water to yield the products **3a-b and 5a-b** (Fig. S1b, S2b), where the concentration of peptides was determined from the area under the RGD-DBCO peak at 220nm from a previously generated HPLC chromatogram calibration curve. The product was subsequently purified using ethanol precipitation (sodium acetate with 95% ultra-pure ethanol followed by 80% ethanol with 30-minute freeze cycles at -20°C and centrifugation at maximal speed for 30 minutes at 4°C). The reaction was quantified using Novex™ TBE-Urea denaturing Gels run on the SureLock™ Mini-Cell System in Novex® TBE Running Buffer (Fig. S5,S16). In order to disrupt secondary structures, the oligos were heated to 90°C for three minutes using a heating block, snap-cooled on ice and loaded into a gel in Novex™ TBE-Urea Sample Buffer. The samples were run for 90 minutes and were stained using SYBR™ Gold Nucleic Acid Gel Stain suitable for visualisation of single-stranded DNA. The gels were visualised using a BioSpectrum Imaging System (UVP) using exposure times from 1 to 8 seconds. The 10/60 ssDNA ladder was used as a control (Fig. S5,S16). Analysis of the bands and peptide conjugation yield calculations were performed using FIJI.

Fabrication of TrAPs for 2D VEGF-aptamer experiments

An aptamer previously found to bind VEGF (K_D=0.2 nM, US Patent 7153948 B2) with an azide on its 5' end and a maleimide on its 3' end was ordered from ATDBio (Apt_VEGF_1, Table S2). The 5' end of 1mM aptamer in DI water was modified with a 3.6-fold molar excess of the DBCO-amine linker dissolved in DMSO. The reaction was left to proceed at 4°C overnight. Next, the 3' end was coupled to either a biologically active peptide or its scrambled counterpart that was added in three-fold molar excess in PBS and was left to react for 3 hours. The peptide sequences we used were c(RGDfC)) (002280V, Peptides

International) and c(RADfC) (001456V, Peptides International), respectively. The coupling between peptides and the aptamer was achieved through a thiol-maleimide reaction following reduction of disulfide bonds between cysteines using TCEP.

MALDI-TOF of TrAPs

Successful conjugation of peptides to the aptamers to form the TrAPs was confirmed by mass spectrometry. A solution of 0.5 mg/ml PDGF-BB RGD-TrAPs was analysed in a 4800 MALDI-TOF-TOF (AB Sciex, CISBIO Mass Spectrometry Core Facility, Imperial College London) using a 3-hydroxypicolinic acid (3-HPA) matrix (0.5 M 3-HPA in a 1:1 solution of 50mM aqueous diammonium and acetonitrile).

Functionalization of 2D coverslips

A Michael addition reaction was employed to graft thiol-terminated biomolecules (cysteine-terminated peptides and thiol-TrAPs) onto maleimide functionalized coverslips (Fig. S8). The coverslips were functionalized based on a protocol described previously [1]. Briefly, the coverslips were first cleaned using piranha etch (70% sulfuric acid, 30% hydrogen peroxide) at 80°C, rinsed in deionized water and dried. Next, amino-groups were introduced using 3-Aminopropyl(diethoxy)methylsilane for 30 minutes, washed with isopropanol, ultrapure water, dried and cured at 80°C for 1 hour. The coverslips were incubated in borate buffer for 1 hour to deprotonate the amino groups and subsequently dried under nitrogen. One side of each coverslip was then marked using a permanent marker, and the other functionalized with maleimide by sandwiching 50 mM maleimide-PEG2- succinimidyl ester solution in borate buffer in between coverslips. Following an hour-long incubation under humidified conditions, the coverslips were cleaned using ultrapure water and dried.

The dithiol groups on the TrAPs were cleaved using soluble 50mM TCEP, the by-product was removed using ethanol precipitation, and the samples dried using vacuum centrifugation at 40°C (miVac, SP Scientific). The peptides were reduced using TCEP reducing gels. The biomolecules at concentrations ranging from 0-100 μM were sandwiched in between two maleimide functionalized surfaces, and the reaction was left to proceed for 1 hour (Fig. S9). The TrAP:peptide molar ratio used for experiments was 1:5, with the exact concentrations used for the original cell adhesion and proof of concept experiments given in Tables S3 and S4. For cell-selective adhesion experiments, 100 μM of either CVAPG, CREDVW, GRGDSPC or GRDGSPC was used. The reaction was carried out in a humidified chamber to prevent the solution from drying out. Care was taken to avoid TCEP traces in the final mixture, as tertiary phosphines react with maleimides and reduce overall coupling efficiency [2]. The pH was buffered using the coupling buffer, as the reaction proceeds approximately 1000 times faster with thiols than amines at neutral pH, but this equilibrium shifts at higher pH values [3].

Fabrication of Maleimide-Thiol polyacrylamide gels

Polyacrylamide gels were fabricated following methods described by Tse and Engler [4]. First, the circular coverslips (1.5 thickness, Agar scientific) were cleaned in subsequent baths in 2-propanol, DI water, 1mM HCl and DI water, each lasting 5-minutes. The coverslips were methacrylated by a 5-minute incubation in a solution of 50μL 3-(Trimethoxysilyl)propyl methacrylate with 300μL of 0.1M acetic acid in 10 mL of ethanol to afford uniform adhesion of the polyacrylamide gel to the coverslips, followed by rinsing with ethanol and drying. The polyacrylamide coating was prepared by mixing acrylamide solution and N,N'-Methylenbisacrylamide solution in the presence of the initiators

N,N,N',N'-Tetramethylethylenediamine and ammonium persulfate. The gels were then left to equilibrate in PBS. The final formulation consisted of 8% acrylamide and 0.48% bis-acrylamide as our initial experiments confirmed that maximal proliferation occurs on surfaces with this composition.

TrAPs and peptides were then coupled to polyacrylamide-functionalized coverslips through their amino-modified 5'end (Fig. S10). The polyacrylamide surface was activated using sulfo-SANPAH exposed to 365-nm UV light for 10 minutes. TrAPs were subsequently added to the gels at 120 μM in 50mM HEPES and incubated overnight at 37°C. This mixture additionally contained a primary amine containing GRGDSPC sequence at 290 μM as it has been previously established that polyacrylamide gels without cell-adhesive peptides do not promote cell adhesion and survival (Fig. S7, Table S5) [4]. The next day, the hydrogels with TrAPs and RGD were rinsed thoroughly with PBS to remove any unbound chemicals.

In addition, we developed a second alternative approach to polyacrylamide functionalization with TrAPs (Fig. S6). This approach utilised the addition of acrylate-PEG-Maleimide into the copolymerisation mixture, which enabled the introduction of thiol-reactive maleimide sites into the gel. The polymerisation mixture was prepared using the following reagents: acrylate-PEG-maleimide (ACRL-PEG-MAL), acrylamide solution (40% w/v, N,N'-Methylenbisacrylamide solution, 2% in DI water), TEMED, and ammonium persulfate. The ACRL-PEG-Mal solution was dissolved in PBS immediately before use to a concentration of 100mM. The final composition of polyacrylamide gels was 8 w/v % acrylamide, 0.48 w/v % bis-acrylamide and 0.17 % w/v ACRL-PEG-Mal. These components were thoroughly mixed using vortex and crosslinked using 1/100 volume of APS and 1/1000 volume of TEMED. Care was taken to keep the thickness of the coatings constant, which was achieved by adjusting the volume of polyacrylamide-maleimide gel precursors depending on coverslip size. For cell adhesion experiments (Fig. S7), functionalization of maleimide-polyacrylamide coverslips was achieved by sandwiching 5 μM thiol-TrAP solution in coupling buffer using the same protocol as for functionalization of maleimide-glass coverslips.

Quantification of surface modification

Three methods were used to quantify the amount of conjugated oligos. First, VEGF-TrAP-modified surfaces were incubated with anti-sense oligos tagged with IRD700 in PBS (Fig. S6) purchased from IDT. The concentration of anti-sense oligos was 1.5 times higher than the concentration of original TrAP incubation solution, with the volume being kept equal. Upon an hour-long incubation, the surfaces were washed three times with PBS. The surfaces were imaged on the Widefield Microscope with LED illumination and Hamamatsu Flash 4 camera (Zeiss Axio Observer inverted microscope) controlled by Zen acquisition software. During acquisition, the settings were adjusted for the TrAP group and were kept constant across groups. Images were subsequently handled in FIJI. Specifically, brightness and contrast were adjusted using the same minimal and maximal brightness values for all images to allow for optimal visualisation of data. In order to correct for uneven illumination, an image of polyacrylamide gels in the red channel that has not been incubated with the antisense strand has been subtracted from all other conditions.

Secondly, the QuantiFluor® ssDNA system was used to quantify aptamer conjugation to polyacrylamide hydrogels via sulfo-SANPAH based on manufacturer's instructions. The fluorescence was read at $492_{exc}/528_{em}$ nm using a plate reader (Fig S10).

The final method to quantify the amount of conjugated oligos utilised SYBRGold (Fig S9). Initial experiments confirmed that the intensity of the signal (measured as mean intensity over a pre-defined area in the program FIJI) increases linearly with increasing SYBRGold concentration. Upon conjugation, the surfaces were incubated with SYBRGold for 20 minutes, dried under the flow of nitrogen, and subsequently imaged using the UVP illuminator. Calibration curves were created by depositing 1 µL drops of varying oligo concentrations on glass slides and rapidly evaporating the solvent on a hot plate.

ELISA

In order to quantify the passive release of PDGF-BB from TrAPs, along with the number of washes are needed to reduce non-specific binding, PDGF-BB in bovine serum albumin (BSA) was incubated with PDGF-BB-TrAP-modified coverslips overnight. The supernatant was subsequently removed and stored at -20°C. To quantify release, incubation media (1% BSA in PBS) was added and collected at regular intervals and stored at -20°C until analysed. The ELISA was conducted based on manufacturer's instructions, with the results read using a microplate reader (SpectraMax M5; Molecular Devices). Prior to analysis, the absorbance readout at 540nm was subtracted from the absorbance readout at 450nm. A four-parameter logistic regression was used to fit the calibration curve data. Subsequently, the obtained concentrations were multiplied by the original dilution factors (either 7.5 or 4). Duplicate technical replicates were averaged.

Functionalization of 3D collagen scaffolds

Commercially available sterile collagen sponges were purchased from Avitene. Circular scaffolds were punched out using 5-mm Miltex biopsy punches. As the manufacturing process leaves collagen scaffolds acidic, scaffolds were incubated in a 0.1M MES: PBS (1:15 parts) buffer to increase the pH. Any bubbles that remained entrapped within the highly porous scaffolds were mechanically removed. For cell-selective TrAP activation experiments, scaffolds were tagged with Texas Red-X succinimidyl ester (18.4 µM in 200 mM phosphate buffer, pH 8.5) for two hours at room temperature under shaking. Then, scaffolds were washed with 0.1M MES:PBS (1:15 parts) buffer three times and subsequently crosslinked and functionalized with maleimide using carbodiimide chemistry (Fig. S13). This was achieved by crosslinking 3.18 mg/ml hetero-bifunctional linker 1-(2-Aminoethyl)maleimide hydrochloride (15 mM) with collagen using 11.5 mg/ml N-Ethyl-N′-(3-dimethylaminopropyl)carbodiimide hydrochloride (EDC) and 5.19 mg/ml N-Hydroxysulfosuccinimide sodium salt (NHS) in 0.1M MES:PBS (1:15 parts) buffer. The EDC/NHS/COOH ratio was kept 5:2:1 as previously described [5]. All of these reagents were added at 150 µL per 5-mm scaffold. The reaction was left to proceed for at least 12 hours at RT under constant shaking, upon which the scaffolds were washed at least 7 times with DI water and sterile PBS. Each wash lasted a minimum of 5 minutes. For control experiments, the maleimide-functionalized collagen scaffolds were reacted with 2µM GRGDSPC with carboxyfluorescein (FAM) on its N-terminus (GenScript, > 95% purity, standard acetate TFA removal), left to react for 4 hours and subsequently washed at least 7 times with PBS. The scaffolds were then imaged at $495_{exc}/520_{em}$ nm using a microplate reader (Fig S13).

To confirm that aptamers attach to scaffolds specifically due to maleimide-thiol coupling throughout the whole scaffold, 5-mm collagen scaffolds were either functionalized with 150 µL of 25µM NHS-maleimide for 1 hour or left unmodified and upon thorough washing incubated with 150 µL of freshly TCEP-reduced 5µM thiol-VEGF-TrAPs **5a** overnight. The scaffolds were subsequently washed at least 7 times in PBS. The scaffolds were then incubated for 1.5hours with 7.5 µM IRD700-antisense strands in PBS, washed twice in PBS

and once in DI water. Finally, the scaffolds were fixed in 4% formaldehyde, washed with PBS, left to permeate with O.C.T, snap-frozen in O.C.T. and stored at -80°C until cryosectioned using a cryostat OTF/AS (Bright Instruments) with a cryosection temperature of -22°C, section thickness 10µm and interval 200 µm. The individual sections obtained from the middle region of the scaffolds were imaged (Fig. S14).

Finally, an optimized functionalization protocol was employed to fabricate PDGF-BB aptamer-modified scaffolds in proof-of-concepts experiments. In these experiments, thiol groups on TrAPs and/or peptides (used in control groups) were reduced as described above and incubated with maleimide-collagen at 1µM and washed 7 times in PBS.

Quantification of cell proliferation based on metabolic activity

The non-destructive, cell-permeable PrestoBlue™ Cell Viability Reagent was used to quantify metabolic activity of cells as a measure of proliferation. This assay is based on the reduction of resazurin to a fluorescent resorufin and the fluorescence signal increases linearly with increasing cell count over a wide range of cells. The assay was read 2 to 4 hours after addition at 1:9 volumetric ratio to media, as specified by the manufacturer, on a FLUOstar microplate reader (BMG LabTech) at $544_{exc}/590_{em}$ nm. Black cell culture microplates with µCLEAR® bottom were used in all experiments to minimize background fluorescence and crosstalk. Prior to analysis, all data were blank corrected. It should be noted that the slope of the signal changes slightly at 5000 cells, so while the data presented here are indicative of differences in cell count, these values are not absolute.

Endothelial cell culture

The cell line, human dermal microvascular endothelial cells 1 (HMEC-1)[6], was a gift from Prof Rob Krams in the Department of Bioengineering at Imperial College London and was tested for mycoplasma infection. The cells were grown in MCDB 131 medium, supplemented with 10 % foetal bovine serum (FBS), 10 mM L-Glutamine, 10 ng/ml Epidermal Growth Factor, 1 µg/mL hydrocortisone and 100 U/ml Pen/Strep.

Prior to conducting control growth factor stimulation and aptamer inhibition experiments, the cells were seeded at 10 000 cells per well and left to adhere overnight, upon which the cells were serum starved for 24 hours in minimal medium (MCDB 131, 0.1% FBS, 10 mM L-Glutamine, 100 U/ml Pen/Strep). Appropriate amounts of recombinant human VEGF-165 (VEGF-165) and aptamers were added to the wells in fresh minimal medium, and the proliferation was analysed continuously using an Incucyte® (EssenBioscience) (Fig. S11) or PrestoBlue™ reagent (Fig S12a).

For proof of concept experiments, the TrAPs were loaded with VEGF-165 by incubating individual TrAP-modified polyacrylamide hydrogels in a 10 ng/ml VEGF-165 PBS solution for 2 hours. The same incubation regime was applied to control hydrogels without TrAPs. The unbound VEGF-165 was then removed by aspirating the incubation medium and rinsing the hydrogels with PBS. Finally, the hydrogels were seeded with HMEC-1 that had been validated to proliferate in a VEGF-165 concentration-dependent manner (Fig S12a). The assay was done in a 96-well plate with a seeding density of 10 000 cells/well and 7 repeats per condition. Cell proliferation was quantified fluorometrically on a plate reader after 24 hours using the PrestoBlue™ assay (Fig. S12b).

Fibroblast cell culture

Primary dermal papillary fibroblasts (HDFs) derived from the scalp of a healthy individual were a gift from Dr Claire Higgins in the Department of Bioengineering at Imperial College London. The cells were maintained in Dulbecco's Modified Eagle Medium, supplemented with 10% FBS, 10 mM L-Glutamine and 100 U/ml Pen/Strep.

In all experiments, the cells were used in between passages 3 and 5. Prior to growth factor treatment with the recombinant human PDGF-BB, the cells were serum starved for at least 24 hours using minimal media (low glucose DMEM, 0.1% FBS, 10 mM L-Glutamine, 100 U/ml Pen/Strep). Cell viability was analysed using PrestoBlue™ Cell Viability Reagent.

For TrAP experiments, the functionalized glass slides were left to incubate with 100 μL of 15ng/ml PDGF-BB overnight. The incubation media were aspirated, and the glass slides washed twice with PBS, each wash lasting 15 minutes at 37°C. The cells were subsequently seeded at 4000 cells per well. Proliferation was analysed on day 2 using a microplate reader following a 2 hour incubation with PrestoBlue™ assay.

Smooth muscle cell culture

Human Aortic Smooth Muscle Cells (HAoSMCs) isolated from the human aorta were purchased from Promocell. The cells were maintained in Smooth Muscle Cell Growth Medium 2.

In all experiments, the cells were used in between passages 3 and 5. Prior to growth factor treatment with the recombinant human PDGF-BB, the cells were serum starved for at least 24 hours using minimal media (Smooth Muscle Cell Basal Medium 2, 1% FBS, 10 mM L-Glutamine, 100 U/ml Pen/Strep).

Quantification of cell-selective adhesion

Prior to conducting cell-selective activation experiments, the differential adhesion properties of HDFs and SMCs was verified by seeding each cell type onto peptide-functionalised (either CVAPG, CREDVW, GRGDSPC or GRDGSPC) 10 mm coverslips at a density of 20000 cells/well. Cells were left to adhere under serum starvation conditions for 24h. Wells were then washed once with PBS to remove loosely attached cells and fresh medium was added before taking representative brightfield images from the centre of each well using an inverted Leica DM IL LED microscope. The number of adherent cells per frame was manually counted from the obtained images. Cells were subsequently fixed and stained for actin cytoskeleton and focal adhesions.

Cell culture in 3D

TrAP-modified scaffolds and RGD-modified control scaffolds (Apt_PDGFBB_1, Table S2,S6) were placed into a 96-well plate and incubated with 100μL of 100 ng/ml PDGF-BB per well overnight. The next day, they were washed twice with PBS. Prior to seeding, the scaffolds were gently blotted.

Prior to the experiment, HDFs at P3 were serum starved for 24 hours. The cells were then seeded at 13 000 cells/well in a total volume of 10μL. Approximately half of the volume was dispensed on top of the scaffolds, which were subsequently turned over and the remaining volume was dispensed onto the reverse side. The scaffolds were subsequently

placed in an incubator for 60 minutes to allow for early adhesions between the cells and scaffolds to form. Finally, 100 µL of minimal medium per well, either with or without PDGF-BB (100 ng/ml) was gently added. The media were changed after 48 hours.

The effect of the growth factor delivery route was assessed after 4 days in culture. As our optimisation experiments confirmed that PrestoBlue™ readouts are unaffected by the highly porous collagen scaffolds, this assay was used to assess metabolic activity. Specifically, the original media was removed and replaced with PrestoBlue solution. The assay was incubated with the scaffolds for 3 hours on an orbital shaker placed inside an incubator (37°C, 5% CO_2) and read using a plate reader.

For cell-selective TrAP activation experiments, TrAP-modified scaffolds (Apt_PDGFBB_2, Table S2,S7) were loaded with PDGF-BB as described above. Experimental groups consisted of scaffolds with either RGD-TrAPs, scr-TrAPs, VAPG-TrAPs, or maleimide scaffolds (without conjugated TrAPs), for both fibroblasts and SMCs (n=5 per group). Prior to the experiment, HDFs and SMCs, both at P4, were serum starved for 24 hours (fibroblasts were starved with 0.1% FBS DMEM medium and SMCs with 1% FBS SMC Basal Medium 2). Cells were seeded at 20000 cells/sponge in a total volume of 20µL, following the same procedure described above with 10 0000 cells per side. Finally, 200µL of basal medium (DMEM or SMC basal medium 2) were added to each well. The media was changed every 3 days during the duration of the experiment (days 3 and 6 for the 1-week experiment and days 3, 6, 9 and 12 for the 2-week experiment).

The effect of the selective growth factor delivery was assessed after 1 and 2 weeks in culture. In order to obtain absolute cell numbers and study the morphology of cells at each experimental endpoint, cells were stained with 5µM CellTracker Green CMFDA Dye for 45 minutes inside an incubator (37°C, 5% CO_2), washed twice with PBS and imaged with Leica DMI 6000 CS Inverted Microscope equipped with HC PL APO CS 10x/0.4 dry objective. Texas Red-X fluorophore from the sponges was excited with 2 mW HeNe laser at 594 nm, and Cell Tracker CMFDA dye was excited with 100 mW Argon laser at 488 nm. Twenty z-series optical sections were collected with a step-size of 10 microns, using the Z-drive motor focus. Z-series are displayed as maximum z-projections using Leica Software and analysed using FIJI.

Stability of TrAPs in 3D collagen sponges

PDGF-BB RGD-TrAPs and scr-TrAPs (Apt_PDGFBB_2, Table S2,S7) were conjugated to crosslinked 3D collagen scaffolds through amine-maleimide linkers and loaded with PDGF-BB as described above. HDFs were seeded on the collagen scaffolds also as described above (20,000 cells/sponge) and allowed to grow in basal medium (DMEM supplemented with 0.1% FBS) for two weeks. Media was changed every 3 days during the duration of the experiment. TrAP integrity was assessed at time 0 and 2 weeks using Cy5-conjugated antisense strands. Briefly, collagen scaffolds loaded with 10 µM TrAPs were incubated for 30 minutes with 20 µM Cy5-antisense strands in PBS, washed three times in PBS and imaged under confocal microscope using a 633 nm laser (Leica DMI 6000 CS Inverted Microscope equipped with HC PL APO CS 10x/0.4 dry objective). Background fluorescence was assessed using collagen scaffolds that had not been incubated with Cy5-antisense strands. Images were analysed using FIJI, and mean intensities at times 0 and 2 weeks measured and analysed for statistical significance.

Stability of TrAPs in conditioned media

PDGF-BB RGD-TrAPs (Apt_PDGFBB_2, Table S2,S7) were diluted in fibroblast-conditioned media to a final concentration of 2 μM and incubated at 37°C for one week. TrAPs were then diluted with DNAse-free water to a concentration of 10 ng/μl and degradation was analysed by TBE-urea gel electrophoresis. Untreated (not incubated in conditioned media) PDGF-BB RGD-TrAPs were used as non-degraded controls.

Staining

Cells were fixed using 4% formaldehyde solution in PBS for 10 minutes, washed with PBS, and permeabilised using 0.1% Triton X-100. The actin cytoskeleton was then stained by incubating cells in Alexa Fluor™ 633 Phalloidin in 1% BSA in PBS for 30 minutes. For cell-selective adhesion experiments, cells were also incubated with purified mouse anti-human paxillin (1:200 in 1% BSA) and Alexa Fluor™ 488-conjugated goat anti-mouse IgG (1:200 in 1% BSA) for 1 hour respectively to stain for focal adhesions. Subsequently, the coverslips were washed twice in PBS, and the nuclei were stained using Hoechst 33342 according to the protocol specified by the manufacturer. The incubation step was conducted in humidified chambers to avoid evaporation. Following staining, the coverslips were washed twice in PBS and once in DI water. Finally the coverslips were mounted on glass slides using VECTASHIELD antifade mounting medium gently cleaned using KimWipes, sealed using nail varnish and stored at 4°C until imaged. In some experiments, the cytoplasm of live cells was stained using cytocompatible CellTracker™ Green CMFDA (5-Chloromethylfluorescein diacetate) Dye, which was added to a T-75 for 20 minutes at a concentration of 5 μM.

Imaging

The surfaces and cells were imaged using the Zeiss Axio Observer inverted microscope with a fully motorised stage controlled by Zen acquisition software. The microscope is equipped with LED light sources from UV to far-red (emission range 665-715nm) and Hamamatsu Flash 4 camera. Bright field images were taken with Leica DMIL LED microscope equipped with Leica DFC295 digital colour camera and LAS V4.9 software. Images from different channels were merged in ImageJ using a Macro developed by the FILM facility at Imperial College London. Figure panels were assembled in Illustrator.

Statistical Analysis

Statistical analysis was conducted using the Software GraphPad Prism 7. The equality of variances was tested using Shapiro-Wilk test. For growth factor experiments, data were normalised using the Normalise function in GraphPad Prism7 between 0 and 100% where the baseline 0% was considered mean proliferation under no growth factor stimulation and 100% mean of maximal growth factor stimulation (positive control) in each experiment unless specified differently in the figure headings. One-way analysis of variance (ANOVA) with a Tukey's multiple comparisons test or *t*-test, where appropriate, were used to analyse the data. One sample was excluded from the analysis of the polyacrylamide gel experiments because cells were seeded on an unmodified side of the polyacrylamide-coated coverslip. Significance was set at $p < 0.05$. Data are summarised as means ± SD.

SUPPORTING FIGURES

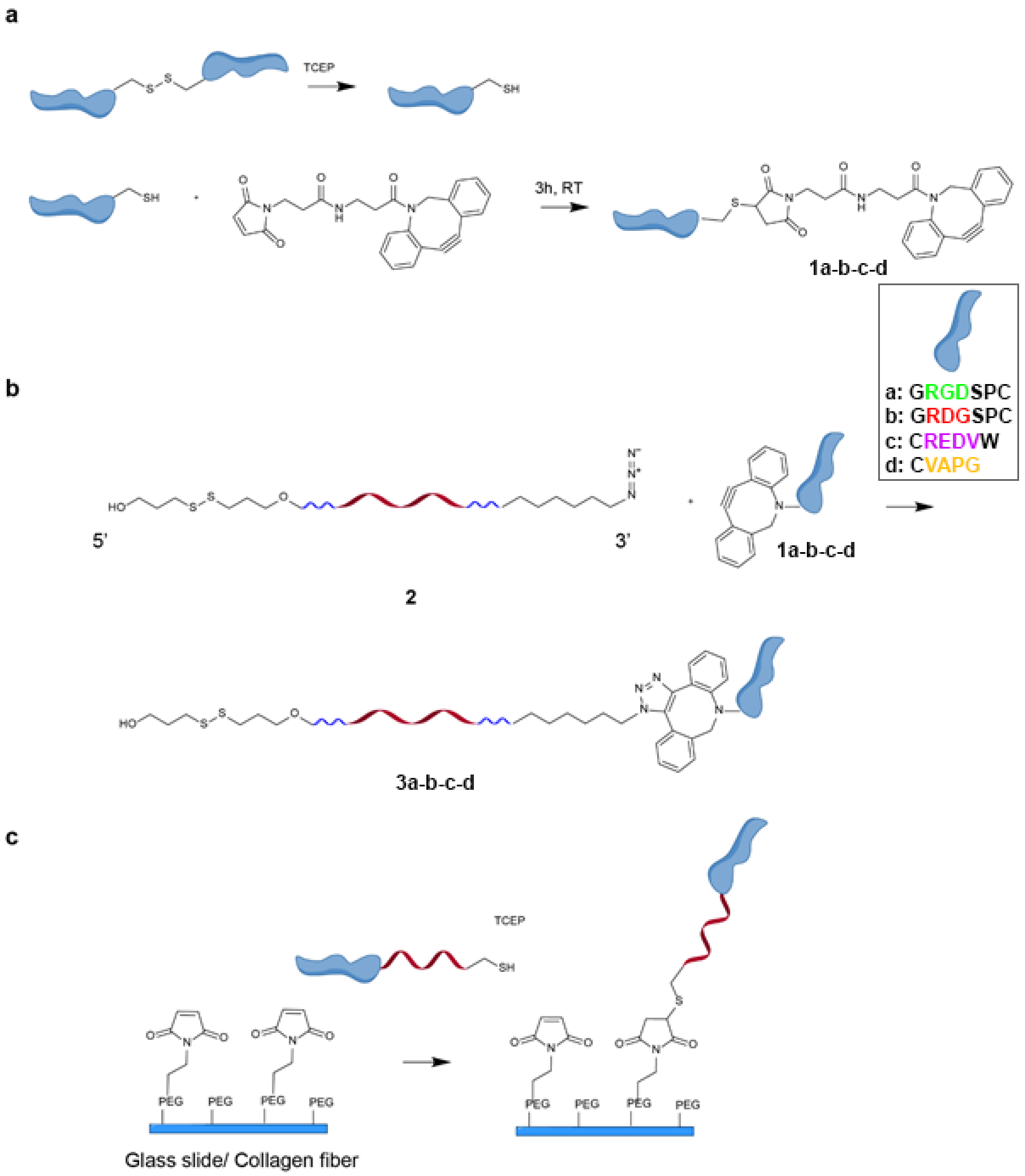

Figure S1. Schematic of the reactions used to prepare TrAPs. (a) GRGDSPC, GRDGSPC, CREDV, and CVAPG were reacted with thiol-DBCO in order to introduce DBCO onto the peptides. (b) PDGF-BB aptamer **2** was reacted with the peptides to create TrAPs. (c) Dithiols were reduced using TCEP and the TrAPs conjugated to a desired material using maleimide-thiol coupling.

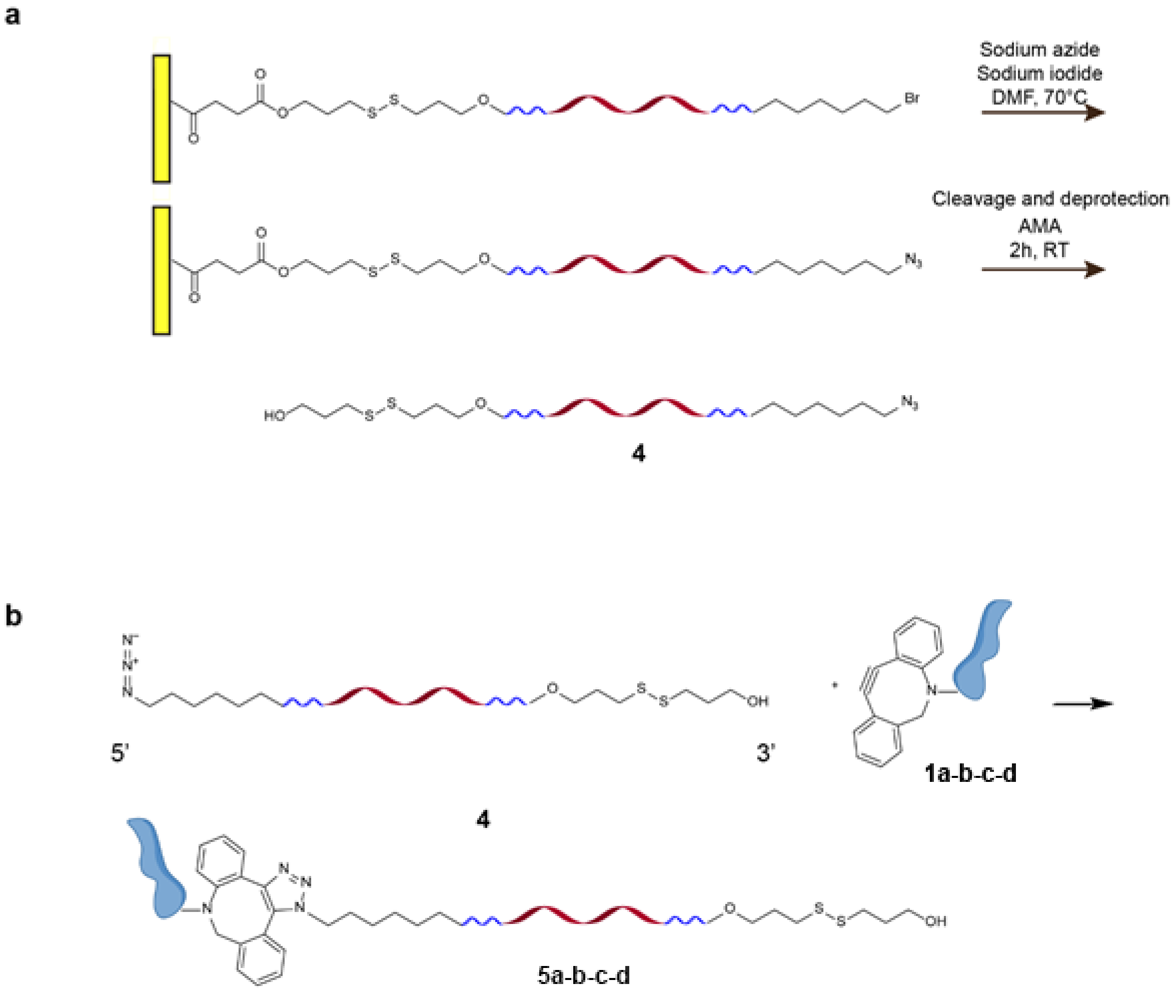

Figure S2. Alternative approach to synthesis of TrAPs. (1) VEGF aptamers **4** were synthesized in-house using a MerMade 6 synthesizer with thiol-modified solid-support and bromine as an end functionality. Bromine was subsequently converted to azide and the oligo cleaved from the solid support. (b) VEGF aptamers were conjugated to peptides **1a-b-c-d** via the 5' end to yield the products **5a-b-c-d**.

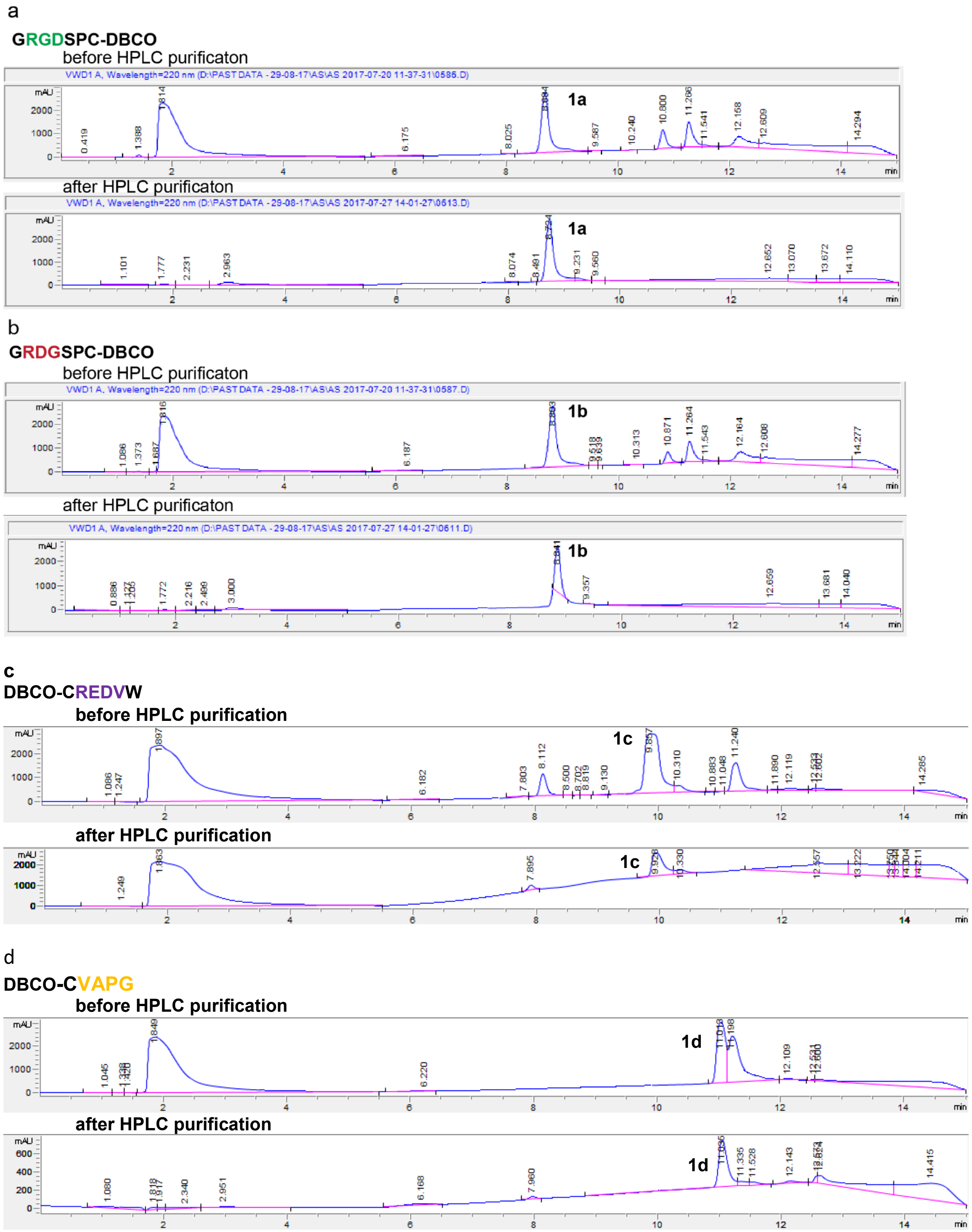

Figure S3. LC-MS demonstrating HPLC purification of DBCO-modified peptides. (a) GRGDSPC-DBCO (**1a**) prior to purification (top) and after purification (bottom). (b) GRDGSPCC-DBCO (**1b**) prior to purification (top) and after purification (bottom). (c) DBCO-REDVW (**1c**) prior to purification (top) and after purification (bottom). (d) DBCO-CVAPG (**1d**) prior to purification (top) and after purification (bottom).

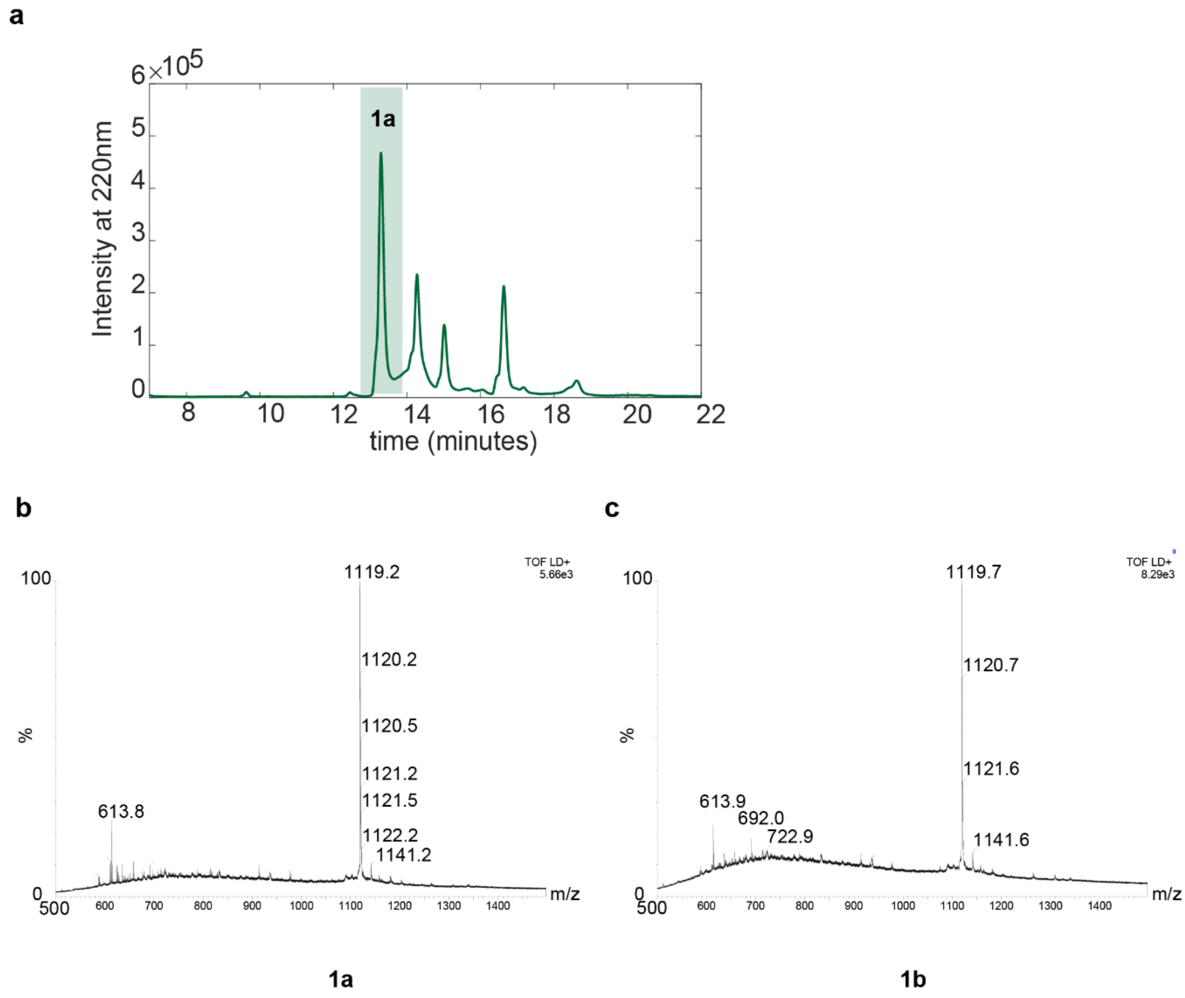

Figure S4. HPLC purification and mass spectra of DBCO-modified peptides. (a) A representative HPLC spectrum of the DBCO-modified peptides **1a** measured at 220 nm. Green background indicates the fraction that was automatically collected and subjected to further analysis (b) MALDI-TOF spectrum of GRGDSPC-DBCO, $MW_{calculated}$ = 1118.19, $MW_{measured}$ =1119.2 $[M+H]^+$ (**1a**) (c) MALDI-TOF spectrum of GRDGSPC-DBCO after HPLC purification (**1b**), $MW_{calculated}$ = 1118.19, $MW_{measured}$ =1119.7 $[M+H]^+$

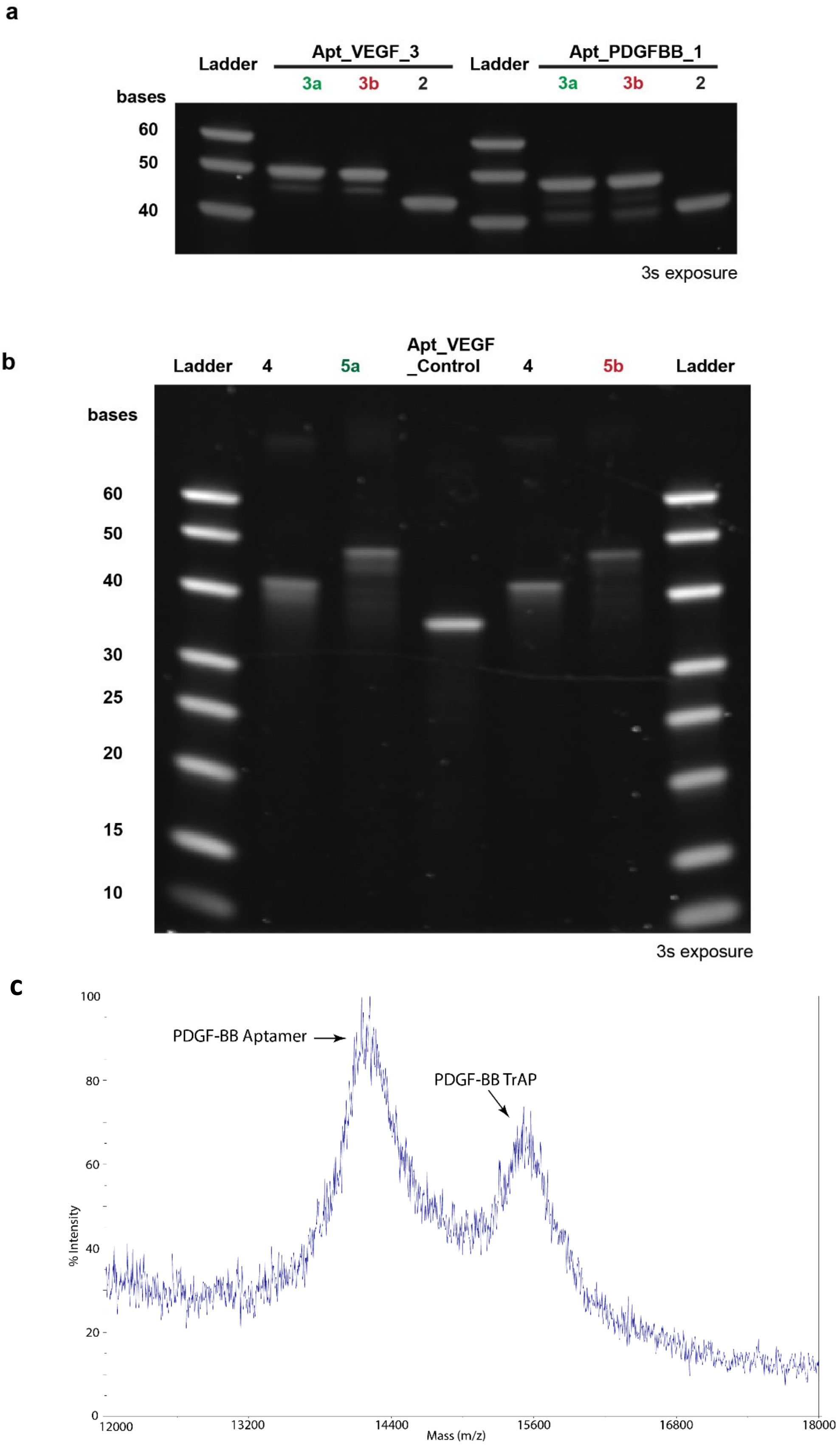

Figure S5. Confirmation of TrAP synthesis via gel electrophoresis and MALDI-TOF. (a) TBE-Urea gels confirmed successful fabrication of both scr-TrAPs (**3b**) and TrAPs. (**3a**) (b) TBE-Urea confirming successful synthesis of anti-VEGF aptamers **(4)** and subsequent fabrication of scr-TrAPs (**5b**) and functional TrAPs (**5a**). (c) MALDI-TOF confirmation of PDGF-BB TrAP (**3a**) fabrication (peptide (**1a**) MW 1118.19 Da).

a

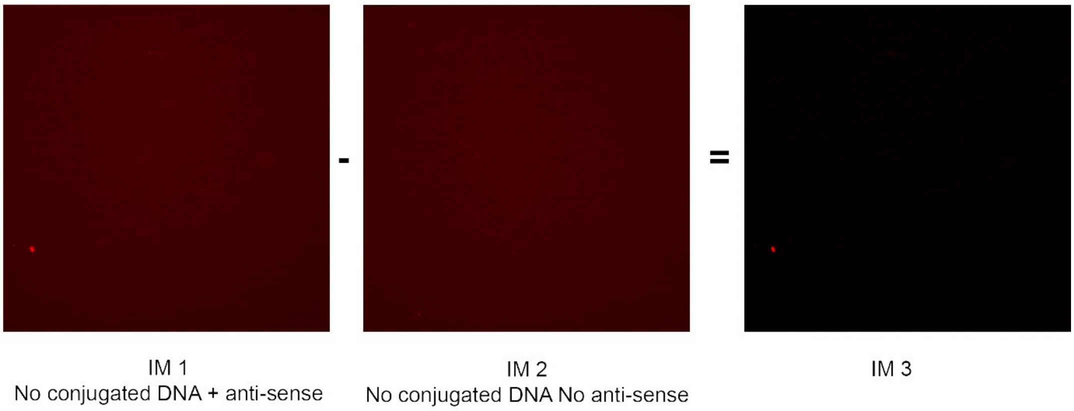

b

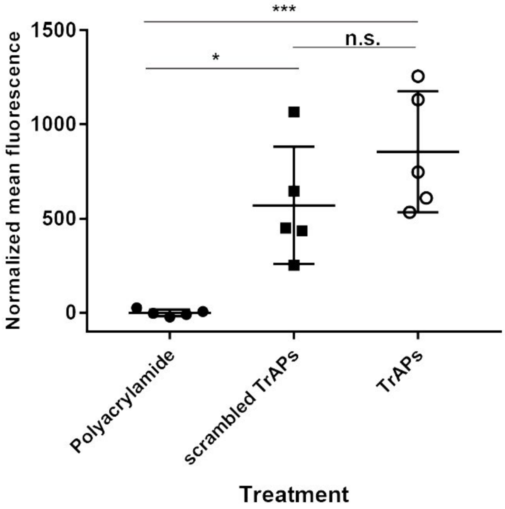

Figure S6: Quantification of aptamer conjugation to 2D polyacrylamide gels. (a) Polyacrylamide gels were incubated with anti-sense IRD700 strand and washed 5 times (IM 1). An image of polyacrylamide gel that has not been incubated with anti-sense DNA and is indicative of polyacrylamide autofluorescence, IM 2, was subtracted from all images to remove background, resulting in IM 3. (b) Quantification of normalized mean fluorescence measured in the far red channel using 5x magnification. Each data point represents mean normalized fluorescence of a whole image taken at 5 different spots from a total of 2 hydrogels per group. Data were analysed using ANOVA, data represent mean ± SD.

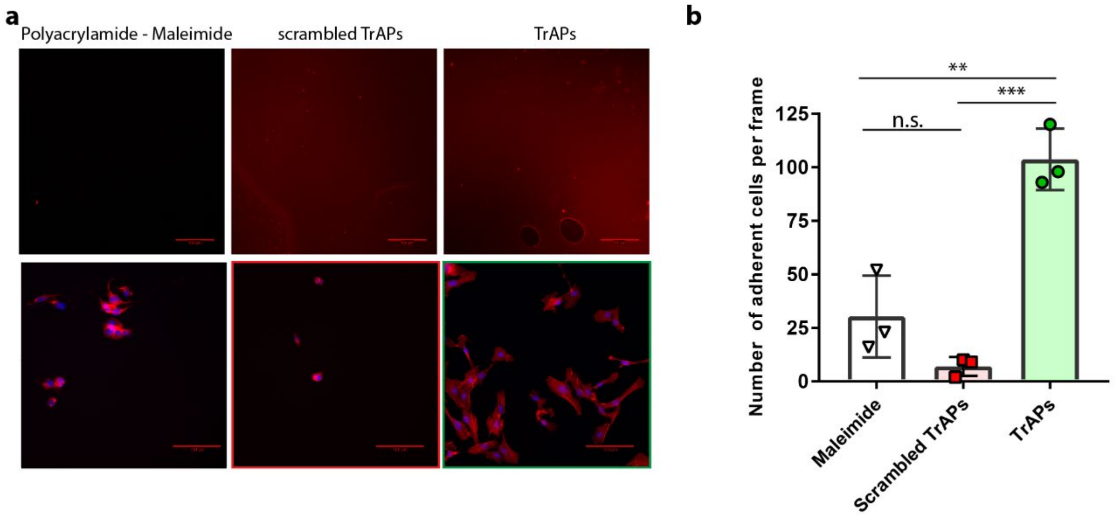

Figure S7. HMEC-1 cells adhere to RGD-containing TrAPs but not to RDG containing scr-TrAPs. (a) HMEC-1 cells adhered to 2D polyacrylamide hydrogels functionalized with anti-VEGF TrAPs (**5a**) but not to scr-TrAPs (**5b**) or maleimide-functionalized surfaces. Scale bar is 500µm for the top row and 100µm for the bottom row. Fluorescent staining is bottom row of actin cytoskeleton (red) cell nuclei (blue). (b) Quantification of the number of adhered cells shows that the number of cells that adhered to TrAPs was significantly higher compared to other surface modifications (n=3, one-way ANOVA, Tukey's multiple comparisons test, **p=0.0017, ***p=0.0004)

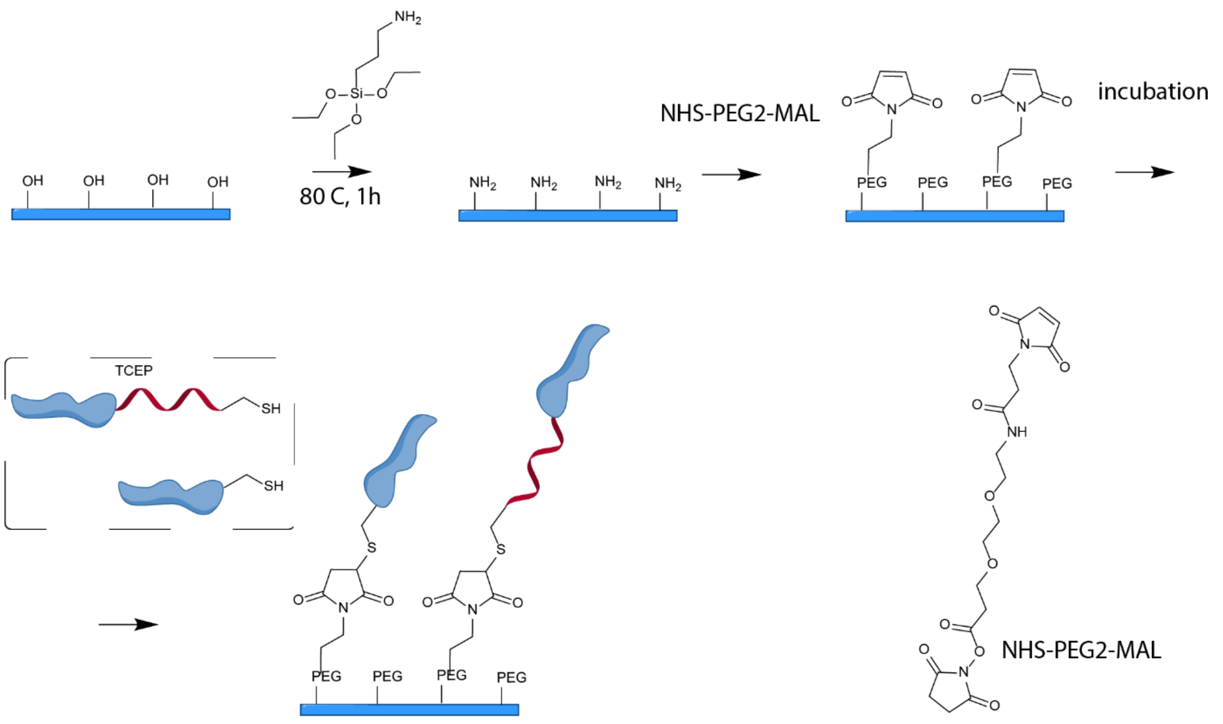

Figure S8. Schematic of glass slide functionalization procedure adapted from Zimmerman and colleagues. [1]

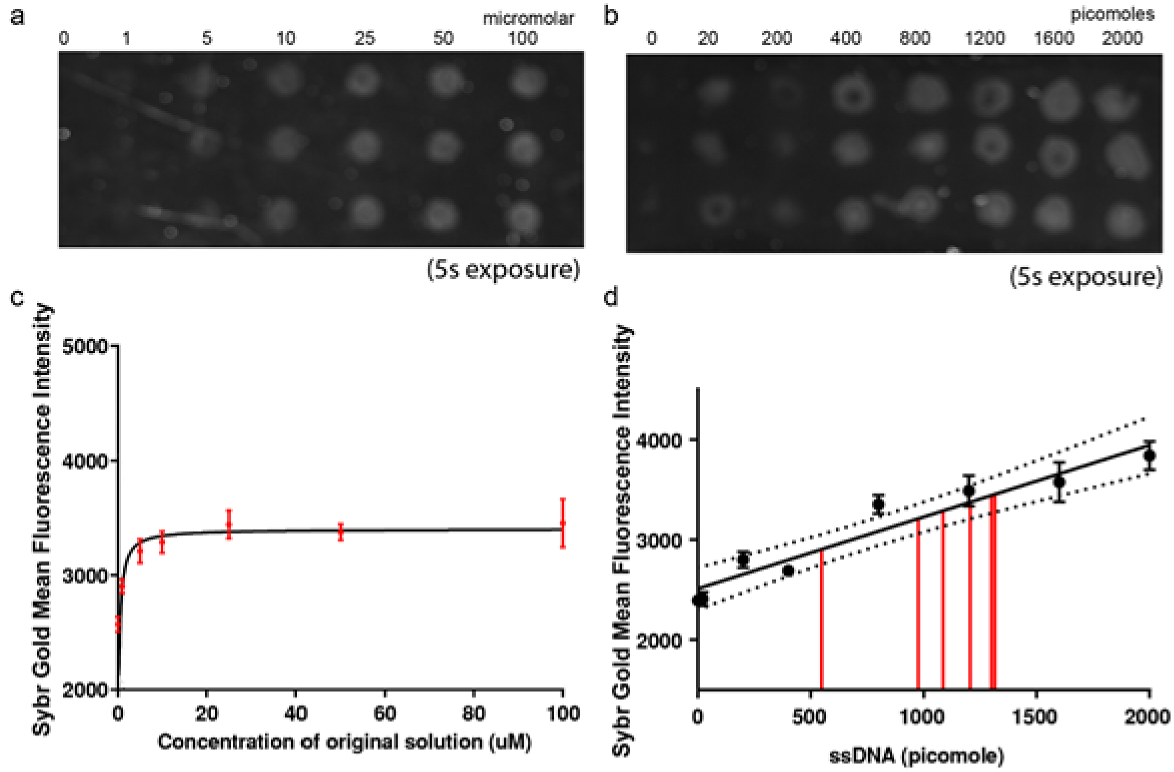

Figure S9. Functionalization of glass slides with TrAPs. (a) 2µl droplets of aptamer solution of varying concentrations were reacted with maleimide-functionalized glass slides for 1 hour, washed, incubated with SYBRGold and imaged under an UVP visualizer using 5s exposure. (b) Calibration curve generated by depositing 2µl droplets of aptamer solution and letting them dry rapidly on a hot plate. SYBRGold was added onto the dried spots and the glass slides were imaged using 5s exposure. (c) Surface coverage with aptamers stops increasing with increasing concentration of the incubation (original) solution of around 20µM, *N*=3. Data represent mean ± SD (red) , black line represents hyberbolic curve fitting ± SE (d) Calibration curve generated in GraphPad prism indicates that each conjugated drop contains between 548.7 (lower limit 431.33, upper limit 655.6) and 1315 (lower limit 1193.0, upper limit 1462.0) picomoles of aptamer. Data points represent mean ± SD (black), interpolation curve represents best-fit values ±SE of linear regression (Y = 0.7166*X + 2512, R^2=0.9265) Red lines represent mean interpolated values.

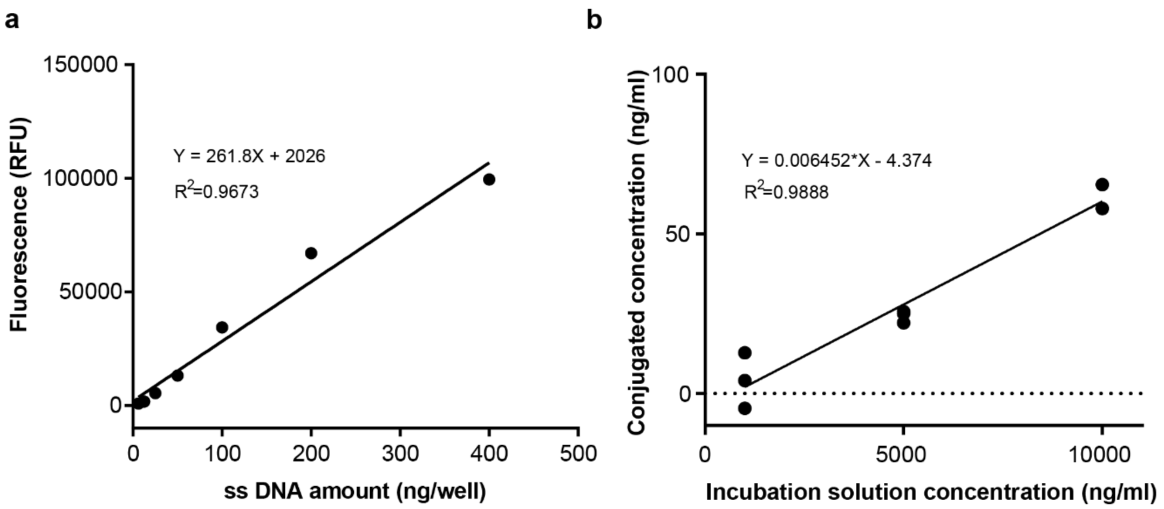

Figure S10. Aptamer coupling to 2D polyacrylamide hydrogels via amine-Sulfo-SANPAH reaction. (a) Calibration curve for ssDNA assay, Promega. Standard was added to blank polyacrylamide-functionalized circular 5mm coverslips. Interpolated curve represents best-fit values of linear regression, n=1,(Y=262.8X + 2026, R^2=0.9673) (b) Confirmation of successful conjugation of amine aptamers to polyacrylamide hydrogels via UV-mediated, sulfo-SANPAH reaction. The amount of conjugated oligonucleotide increases with increasing concentration of incubation solution. *N*=3, only 2 coverslips were preserved for the 10000 ng/ml incubation group. Individual data points are plotted. Interpolated curve represents best-fit values of linear regression (Y=0.006452X-4.374, R^2=0.9888)

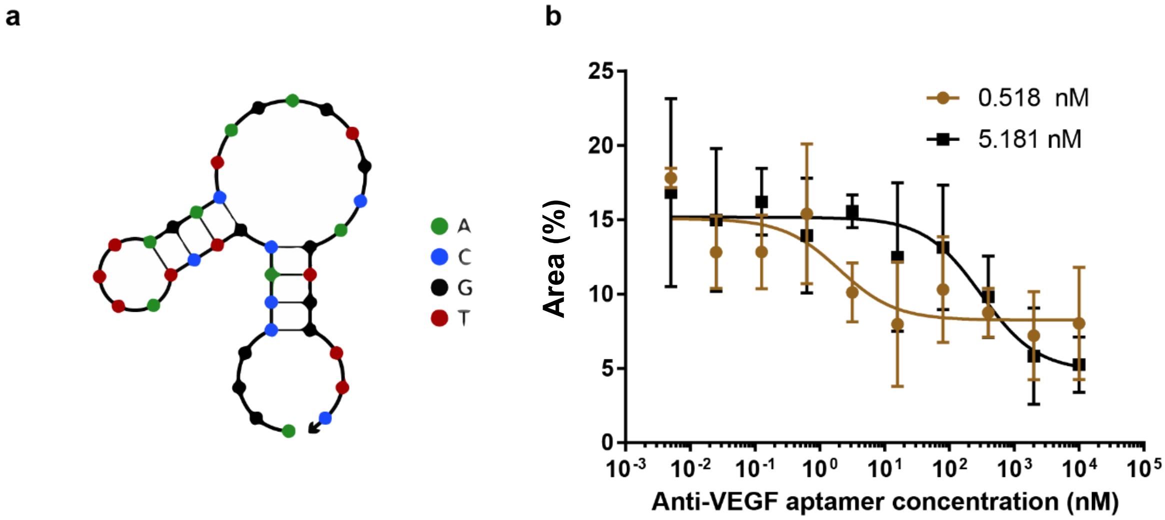

Figure S11. Evaluation of anti-VEGF aptamer's capacity to inhibit VEGF-mediated proliferation. (a) Secondary structure of anti-VEGF aptamer used in this study generated using NUPACK. (b) Inhibition curves for two concentrations of VEGF (0.518 nM, brown, 5.181 nM, black) shows that HMEC-1 proliferation decreases with increasing anti-VEGF aptamer concentration, which suggests that VEGF aptamer inhibits VEGF-165-mediated proliferation. *N*=4. The proliferation (y axis) is indicative of confluence and was determined using an automated software Incucyte from images obtained after 24 hours. The data were fitted with three parameter nonlinear regression.

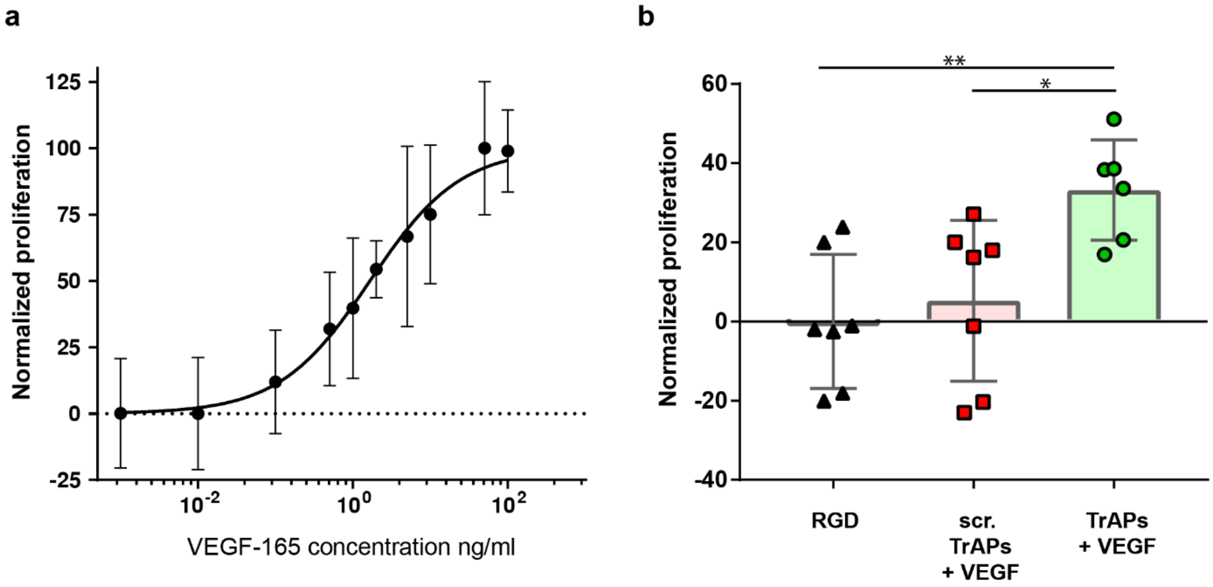

Figure S12. Proof of concept experiment using HMEC-1 cells on VEGF-loaded TrAPs (c(RGDfC) terminated) attached to 2D polyacrylamide surfaces**.** (a) HMEC-1 proliferation increased with increasing VEGF concentration. *N*=7. Black line represents a three parameter best-fit , data are shown as mean values ± SD.(b) HMEC-1 cells on 2D polyacrylamide surfaces modified with TrAPs loaded with VEGF-165 proliferated significantly more compared with 2D scr-TraPs (c(RADfC) terminated**)**-modified loaded surfaces and unloaded 2D RGD-modified surfaces. (*N*=7) (**p*=0.0240, ***p*=0.0076))

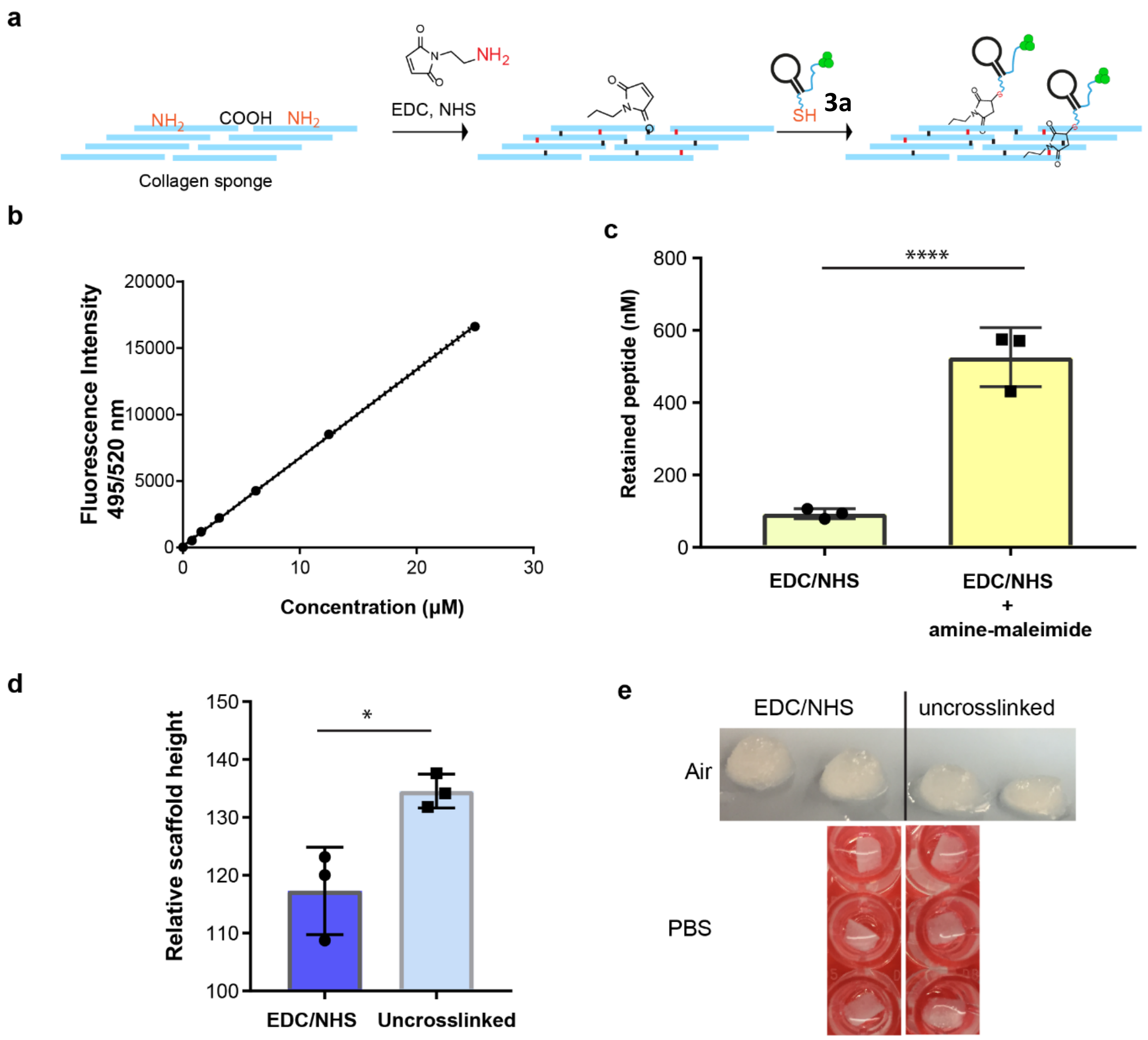

Figure S13. Development of TrAP-collagen functionalization approach. (a) Commercially available collagen sponges were crosslinked and modified with maleimide through incubation of the scaffolds with amine-maleimide in the presence of EDC and NHS overnight. Upon thorough washing, the scaffolds were reacted with TrAPs (**3a**). (b) FAM-GRGDSPC was used to optimize the reaction scheme and FAM fluorescent intensity increases linearly with its increasing concentration across the used range of concentrations. *N*=1, black line represents linear regression fit. (Y = 663.5*X + 99.02, R^2=0.9998) (c) Scaffolds containing maleimide retained significantly higher (*t* test) amounts of FAM-GRGDSPC compared with unmodified, cross-linked scaffolds. (d) The use of EDC/NHS significantly reduced swelling of the scaffolds in PBS solution, measured as height in pixels (*t* test). (e) The EDC/NHS scaffolds retained their shape after removal from PBS to the air while the uncrosslinked scaffolds collapsed. When in PBS, the swelling of uncrosslinked scaffolds was more prominent. Collectively, these morphological changes provide evidence that the crosslinking reaction proceeded as expected [7].

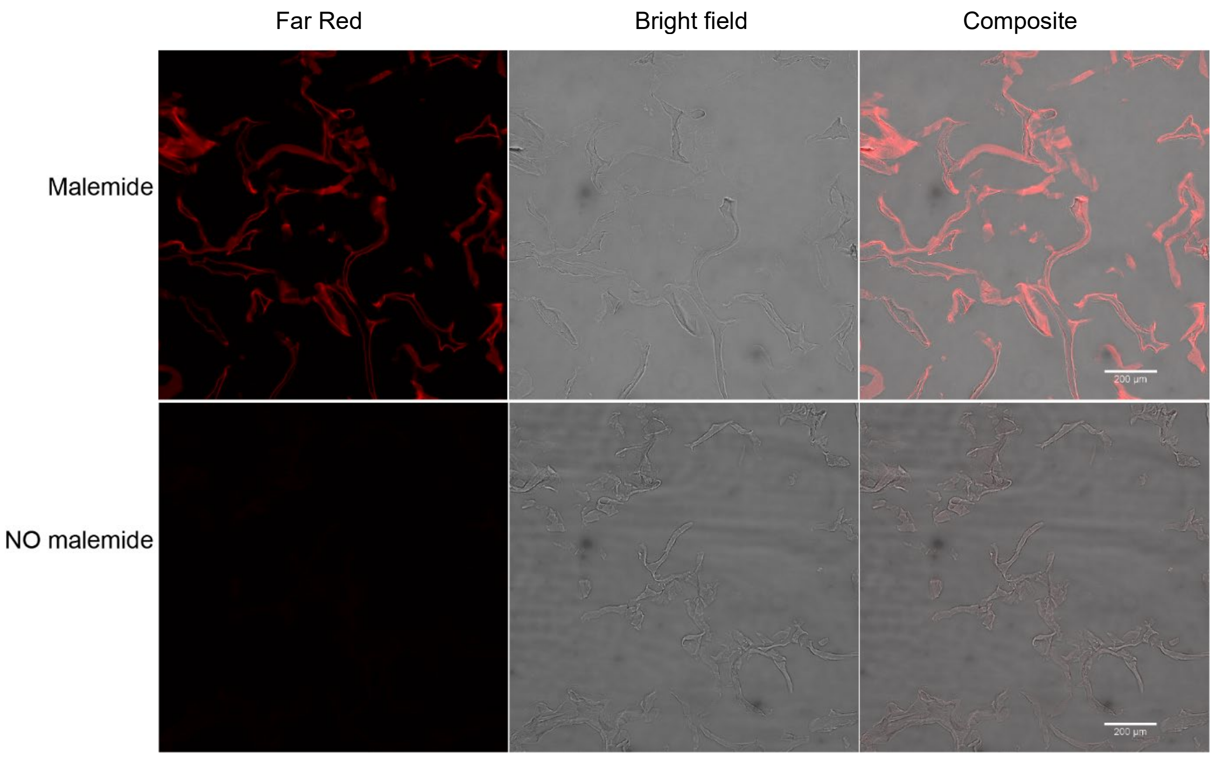

Figure S14. Confirmation that TrAPs react with maleimide throughout the thickness of the scaffolds. Maleimide-functionalized sponges were reacted with VEGF-TrAPs **5a**. The scaffolds were subsequently snap-frozen and sectioned. Individual sections were incubated with IRD-700 tagged anti-sense aptamer, washed thoroughly, and imaged using the same settings across all conditions. The IRD700 aptamer could be detected only on scaffolds pre-functionalized with maleimide. Several images were taken and the images above are representative of each condition. Scale bar is 200µm

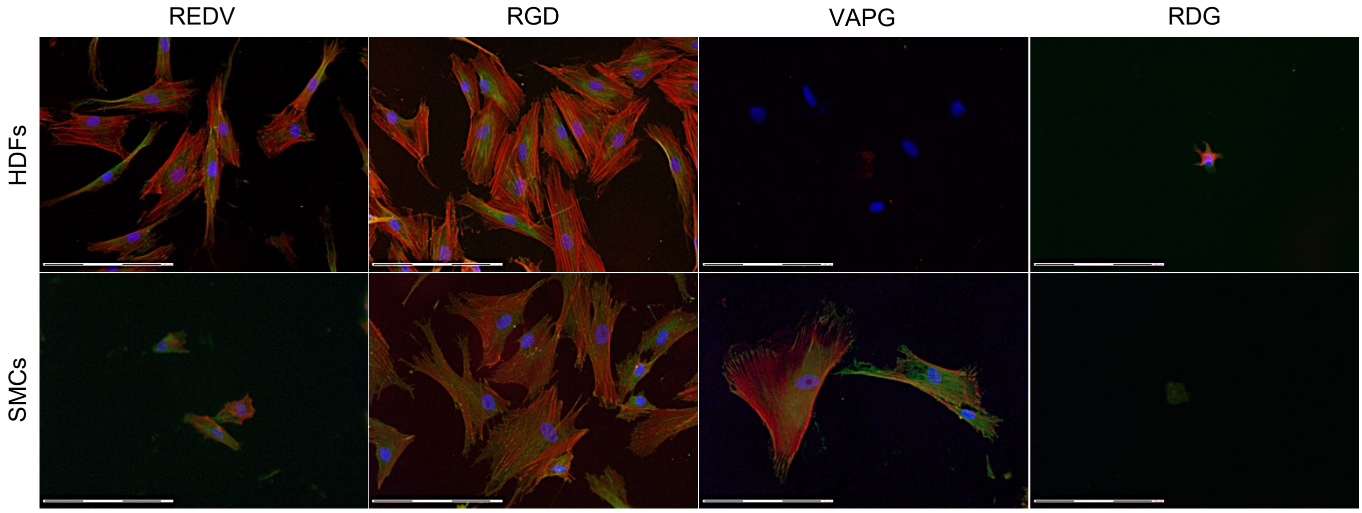

Figure S15. Differential adhesion properties of HDFs and SMCs on peptide-functionalised coverslips. Fluorescent staining of actin cytoskeleton (red), focal adhesions (green) and cell nuclei (blue) confirm that whilst HDFs and SMCs display similar adhesion characteristics on REDV, RGD and RDG-functionalised surfaces, only SMCs are capable of attaching to VAPG surfaces with elongated morphologies indicative of an ability to exert traction forces. Scale bars; 150 µm.

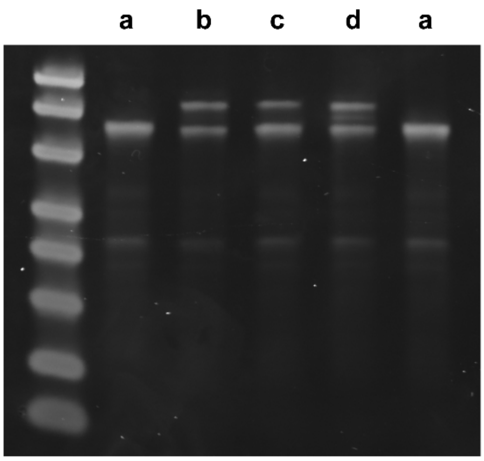

Figure S16. Confirmation of TrAP synthesis for cell-selective activation experiments. TBE-Urea gels confirmed successful fabrication of (a) PDGF-BB polyT aptamer, (b) RGD – TrAPs, (c) scr.RGD -TrAPs and (d) VAPG - TrAPs, with an approximate yield of 50%, which was used as a concentration correction factor during TrAP conjugation to collagen scaffolds in 3D cell-selective-activation experiments.

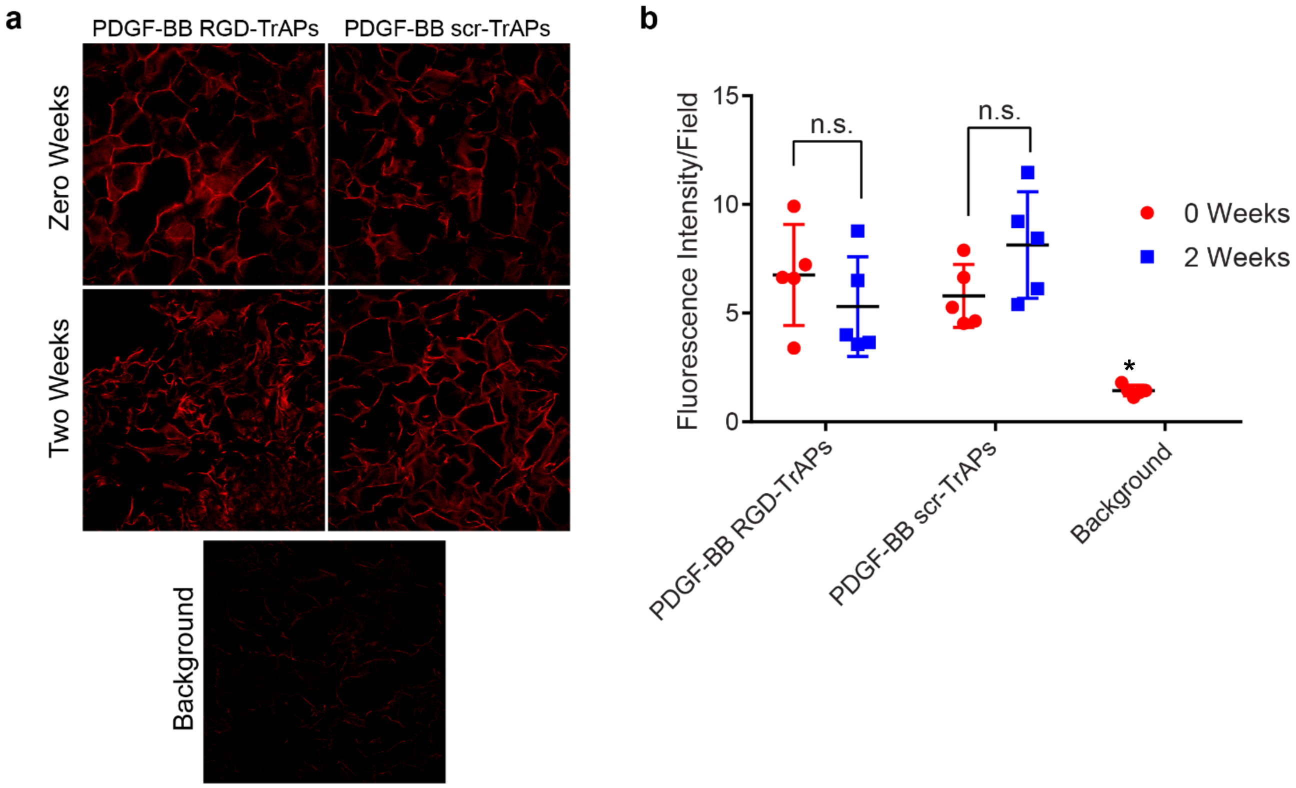

Figure S17. TrAPs do not display significant degradation during two weeks of cell culture with primary human fibroblasts when attached to collagen sponges. (a) Fluorescent images of collagen sponges functionalised with TrAPs and visualised using fluorescently tagged complimentary oligonucleotides immediately following fabrication and after two weeks of cell culture. (b) Quantification of the fluorescence intensity. * $p<0.05$ compared to all experimental groups.

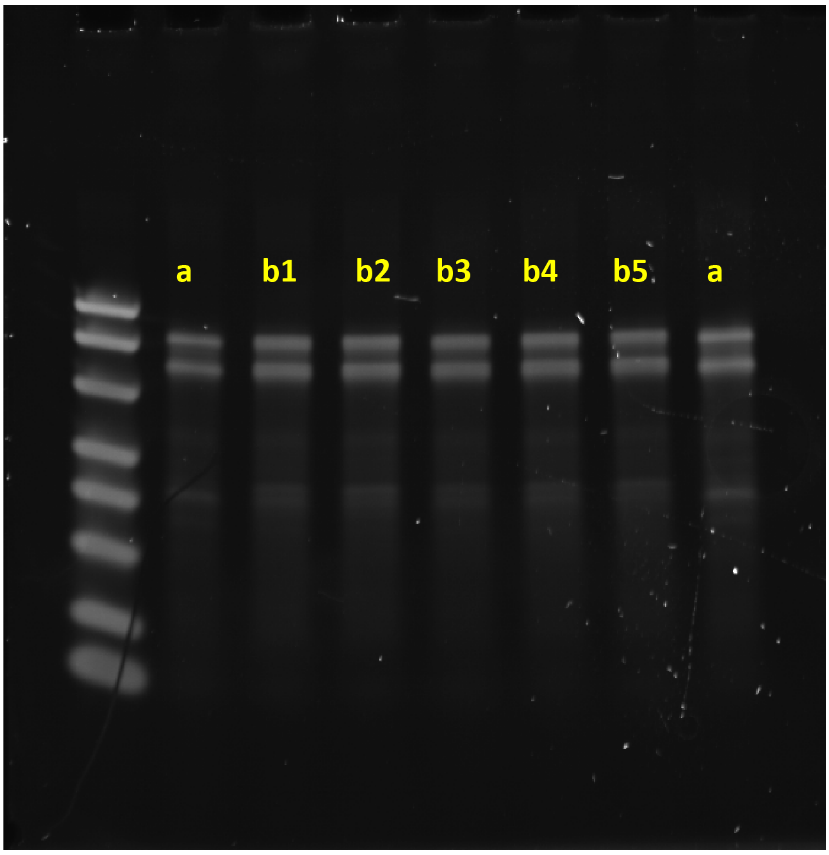

Figure S18. TrAPs do not display significant degradation following one week of incubation at 37°C in fibroblast-conditioned media. (a) TrAPs prior to incubation. (b1-b5) TrAPs following one week of incubation. Upper band is TrAPs with RGD attached while lower band is TrAPs without RGD attached, demonstrating stability of the oligonucleotide with and without a bound peptide.

Supporting Tables

Table S1. Full sequences of the anti-VEGF and anti-PDGF-BB aptamers used in this study. K_D signifies binding constant, MT melting temperature, N number of nucleotides and Ref. Reference

	Oligo (5'->3')	K_D	MT	N	Ref.
**VEGF**	A GGG CCA CGT CTA TTT AGA CTA GAG TGC AGT GGT TC	**0.2 nM**	65.1 °C	**36**	**US Patent 7153948 B2**
**PDGF-BB**	C AGG CTA CGG CAC GTA GAG CAT CAC CAT GAT CCT G	**0.1 nM**	67.3 °C	**35**	[8], [9]

Table S2. Modifications that were introduced onto individual aptamers used in this study. MW stands for molecular weight

ID	Sequence 5'-> 3'	Supplier	MW
**Apt_VEGF_control**	A GGG CCA CGT CTA TTT AGA CTA GAG TGC AGT GGT TC	IDT	11131.2
**Apt_VEGF_1**	Z-A GGG CCA CGT CTA TTT AGA CTA GAG TGC AGT GGT TC-X<br>Z = amino peg4+azido hexanoic acid and X = amino C7+peg8 maleimide	ATDBio	12307.0
**Apt_VEGF_2**	/5azide//isp18/A GGG CCA CGT CTA TTT AGA CTA GAG TGC AGT GGT TC/isp18/ /3thiol-thiol/	Synthesized	12269.3
**Anti_Sense_VEGF**	/5IRD700/GA ACC ACT GCA CTC TAG TCT AAA T	IDT	8034.7
**Apt_PDGFBB_1**	/5thiomc6-d//isp18/C AGG CTA CGG CAC GTA GAG CAT CAC CAT GAT CCT G/isp18//3aziden/	IDT	12093.3
**Apt_PDGFBB_2**	/5thiomc6-d/TTT TTC AGG CTA CGG CAC GTA GAG CAT CAC CAT GAT CCT GTT TTT/3aziden/	Synthesized	14127.3

Table S3. PDGF-BB TrAP, GRGDSPC and GRDGSPC concentrations used in the cell adhesion 2D studies.

	TrAPs	Scr-TrAPs	TrAPs+RGD	Scr-TrAPs + RDG	Maleimide
**PDGF-BB TrAPs**	20 μM		5μM	-	-
**Scr. PDGF-BB TrAPs**	-	20 μM	-	5μM	-
**GRGDSPC**	-	-	20 μM	25 μM	-
**GRDGSPC**	-	-	5μM	-	-

Table S4. PDGF-BB TrAP, GRGDSPC and GRDGSPC concentrations used in the 2D proof of concept studies. Note that the RGD concentrations across groups are matched and TrAPs groups contains additional GRGDSPC and GRDGSPC peptides

	TrAPs group	Scr-TrAPs group	Control Groups
**PDGF-BB TrAPs**	5μM	-	-
**Scr. PDGF-BB TrAPs**	-	5μM	-
**GRGDSPC**	20 μM	25 μM	25μM
**GRDGSPC**	5μM	-	5 μM

Table S5. VEGF-TrAP terminated with either c(RGDfC)) or c(RADfC)) and GRGDSPC peptide concentrations used in the 2D HMEC-1 proof of concept study.

	TrAPs group	Scr-TrAPs group	Control Groups
**VEGF TrAPs**	120 µM	-	-
**Scr. VEGF TrAPs**	-	120 µM	-
**GRGDPSC**	290 µM	290 µM	290 µM

Table S6. PDGF-BB-TrAP and peptide concentrations used to functionalize collagen sponges for a 3D proof of concept study.

	TrAPs group	Scr-TrAPs group	Control Groups
**PDGF-BB TrAPs**	1 µM	-	-
**Scr. PDGF-BB TrAPs**	-	1 µM	-
**GRGDSPC**	-	-	1 µM

Table S7. PDGF-BB-TrAP concentrations used to functionalize collagen sponges for the 3D cell-selective activation study.

	TrAPs group	Scr-TrAPs group	VAPG-TrAPs group	Control Groups
**PDGF-BB TrAPs**	1 µM	-	-	-
**Scr. PDGF-BB TrAPs**	-	1 µM	-	-
**PDGF-BB VAPG TrAPs**	-	-	1 µM	-

Supporting References

[1] J. L. Zimmermann, T. Nicolaus, G. Neuert, K. Blank, *Nat. Protoc.* **2010**, *5*, 975.

[2] E. B. Getz, M. Xiao, T. Chakrabarty, R. Cooke, P. R. Selvin, *Anal. Biochem.* **1999**, *273*, 73.

[3] Greg T. Hermanson, *Bioconjugate Techniques*, Elsevier, **2013**.

[4] J. R. Tse, A. J. Engler, *Curr. Protoc. Cell Biol.* **2010**, *47*, 10.16.1.

[5] M. G. Haugh, C. M. Murphy, R. C. McKiernan, C. Altenbuchner, F. J. O'Brien, *Tissue Eng. Part A* **2011**, *17*, 1201.

[6] E. W. Ades, F. J. Candal, R. A. Swerlick, V. G. George, S. Summers, D. C. Bosse, T. J. Lawley, *J. Invest. Dermatol.* **1992**, *99*, 683.

[7] J. M. Wu, Y. Y. Xu, Z. H. Li, X. Y. Yuan, P. F. Wang, X. Z. Zhang, Y. Q. Liu, J. Guan, Y. Guo, R. X. Li, H. Zhang, *J. Mater. Sci. Mater. Med.* **2011**, *22*, 107.

[8] C.-C. Huang, Y.-F. Huang, Z. Cao, W. Tan, H.-T. Chang, *Anal. Chem.* **2005**, *77*, 5735.

[9] L. S. Green, D. Jellinek, R. Jenison, a Ostman, C. H. Heldin, N. Janjic, *Biochemistry* **1996**, *35*, 14413.
